# Visual and Equipment-Minimized Detection of Avian Metapneumovirus Subtype C by RT-RPA-CRISPR/Cas12a Coupled with Lateral Flow Dipstick

**DOI:** 10.3390/ani16193154

**Published:** 2026-10-08

**Authors:** Qizhang Liang, Shoukang Ma, Hongxing Li, Weiwei Wang, Qiuling Fu, Nansong Jiang, Rongchang Liu, Guanghua Fu, Hongmei Chen, Longfei Cheng, Chunhe Wan, Yu Huang

**Affiliations:** 1 Fujian Academy of Agricultural Sciences/Fujian Provincial Key Laboratory for Avian Diseases Control and Prevention, Institute of Animal Husbandry and Veterinary Medicine, Fuzhou 350013, China; qzl1181@zuaa.zju.edu.cn (Q.L.);; 2 College of Animal Sciences, Fujian Agriculture and Forestry University, Fuzhou 350002, China; 3 College of Life Sciences, Fujian Agriculture and Forestry University, Fuzhou 350002, China

**Keywords:** avian metapneumovirus, RPA, CRISPR/Cas12a, LFD, on-site detection

## Abstract

Avian metapneumovirus (aMPV) is the main etiological agent of turkey rhinotracheitis, causing respiratory distress and economic losses in turkeys. While its impact in broilers and laying hens is relatively limited, aMPV subtype C (aMPV/C) is an important pathogen in ducks and waterfowl, leading to respiratory distress and severe egg-drop syndrome. To overcome the limitations of current diagnostics, such as heavy reliance on laboratory infrastructure and prolonged turnaround times, we established a rapid detection assay integrating recombinase polymerase amplification (RPA) with CRISPR/Cas12a and a lateral flow dipstick (LFD), targeting the conserved *N* gene of aMPV/C. Reaction parameters were systematically optimized, with 30 nmol/L Cas12a and 40 nmol/L crRNA identified as the optimal concentrations. Rigorous specificity evaluation against seven common avian pathogens revealed no cross-reactivity. The limit of detection (LOD) was determined to be as low as 1.3 × 10^1^ copies/μL, outperforming conventional PCR. Validation using clinical samples demonstrated a 98.85% concordance rate with quantitative PCR (qPCR). This platform enables visual detection of aMPV/C within 1 h with minimal equipment requirements. Combining high sensitivity, absolute specificity, and operational simplicity, this assay shows promise as a practical tool for point-of-care diagnostics and epidemiological surveillance of aMPV/C.

## Simple Summary

Avian metapneumovirus, particularly the C type, causes severe respiratory disease and egg drop in ducks, leading to significant economic losses in the poultry industry. Current diagnostic methods rely on laboratory-based tests that require expensive equipment and take several hours, making them impractical for use on farms. To address this problem, we developed a rapid, equipment-minimizing detection method that combines a fast nucleic acid amplification technique with a gene-targeting system and a simple paper strip for visual result reading. Using samples collected from ducks, the method produced results within one hour using only a basic heating device, and a colored line on the strip indicated a positive infection. When compared with the most reliable laboratory test currently available, our method showed 98.85% agreement. This approach offers a practical option for near-farm testing and has the potential to help farmers and veterinarians quickly identify infections, enabling timely intervention and reducing economic losses. Future improvements, such as dry-format reagents that do not require cold storage, will further advance its use in field settings.

## 1. Introduction

Avian metapneumovirus (aMPV), a member of the genus Metapneumovirus within the subfamily Pneumovirinae of the family Paramyxoviridae, is an enveloped, negative-sense, single-stranded RNA virus with a genome of approximately 13.5 kb [1]. The genome encodes eight structural proteins: nucleoprotein (N), phosphoprotein (P), matrix protein (M), fusion protein (F), second matrix protein (M2), small hydrophobic protein (SH), attachment glycoprotein (G), and RNA-dependent RNA polymerase (L) [2]. Based on the genetic heterogeneity of the G gene, aMPV is classified into four subtypes (A–D). Subtypes A and B are distributed globally, while subtype D has only been reported in France [3]. Subtype C, first isolated from turkeys in the United States in 1997, has since been identified in French Muscovy ducks, Korean pheasants, and Chinese broilers and Muscovy ducks [4,5]. In China, aMPV/C was first isolated from broilers in 2012 [6] and was subsequently confirmed to cause egg drop and dyspnea in Muscovy ducks in 2014 [7]. As a highly contagious pathogen, aMPV/C infects various avian species—including chickens, turkeys, ducks, and pigeons—causing respiratory distress, head swelling, and decreased egg production, thereby imposing significant economic burdens on the global poultry industry [8,9,10].

Despite the availability of several detection methodologies for aMPV/C, a critical gap remains in point-of-care diagnostics. Current gold standards-including viral isolation, immunological assays, and conventional PCR are hampered by inherent limitations [11]. Viral isolation is notoriously slow (48–72 h) and labor-intensive. Immunological methods often fail to distinguish between active infection and past exposure. Meanwhile, PCR, although specific, requires expensive thermal cyclers and highly trained personnel, rendering it impractical for field deployment in resource-limited regions [12,13,14].

Consequently, isothermal amplification techniques have emerged as superior alternatives. However, existing methods such as Loop-mediated Isothermal Amplification (LAMP) suffer from complex primer design and the risk of non-specific amplification, which hinders their reliability in field applications [15]. Similarly, Recombinase Polymerase Amplification (RPA) offers rapid amplification but lacks the sequence specificity required to rule out false positives, limiting its standalone clinical utility [16,17]. Therefore, there is a pressing need to develop a rapid, sensitive, and equipment-minimized assay suitable for on-site surveillance.

CRISPR/Cas12a-based detection platforms have rapidly emerged as a transformative tool in molecular diagnostics, primarily due to their high sequence specificity [18]. Mechanistically, the system relies on crRNA-guided recognition of a double-stranded DNA target harboring a protospacer adjacent motif (PAM), which initiates robust Cas12a activation [19]. Upon activation, the enzyme exhibits indiscriminate collateral cleavage activity against surrounding single-stranded nucleic acids, including fluorophore-quencher-labeled reporters, thereby transducing the target recognition event into a measurable fluorescent signal [20,21].

The strategic coupling of RPA with CRISPR/Cas12a technology creates a powerful synergistic effect. This integration not only preserves the rapid kinetics of isothermal amplification but also effectively compensates for RPA’s inherent propensity for nonspecific amplification, significantly elevating diagnostic accuracy [17,22]. Furthermore, by incorporating lateral flow dipstick (LFD) technology, the detection module of this assay has been successfully translated into a field-deployable format. The platform delivers rapid, sensitive, and visually interpretable results, effectively bridging the gap between laboratory-based molecular diagnostics and point-of-care testing requirements [23,24].

In this study, we systematically evaluated various combinations of RPA primers and crRNAs targeting the conserved *N* gene of aMPV/C to establish an optimized RT-RPA-CRISPR/Cas12a-LFD assay. Following meticulous optimization of reaction parameters-specifically determining 37 °C for 40 min with 30 nmol/L Cas12a and 40 nmol/L crRNA as optimal-we rigorously assessed the analytical sensitivity and specificity. Crucially, when applied to clinical samples, our assay demonstrated a 98.85% concordance rate with conventional qPCR. Requiring only a simple heating block and LFD, this platform delivers visual results within 1 h and holds promise as a basis for future point-of-care applications in the diagnosis and epidemiological surveillance of avian metapneumovirus.

## 2. Materials and Methods

### 2.1. Clinical Sampling and Nucleic Acid Extraction

A total of eighty-seven clinical samples, including oropharyngeal swabs, cloacal swabs, and tissue specimens (lung, trachea, and oviduct), were collected from Muscovy ducks (*Cairina moschata*) on commercial duck farms located in Putian, Zhangzhou, and Longyan of Fujian Province, China. The ducks exhibited clinical signs consistent with aMPV/C infection (respiratory distress, nasal discharge, and reduced egg production). All samples were transported to our laboratory under cold-chain conditions for subsequent RNA extraction and analysis. Total nucleic acids were isolated utilizing the Animal Total RNA/DNA Isolation Kit, which was commercially acquired from TianLong Biotechnology (Suzhou, China).

### 2.2. Reagent Procurement and Positive Control Construction

The core RPA kit and LFD were sourced from EZassay Co., Ltd. (Shenzhen, China). The LbCas12a endonuclease (derived from Lachnospiraceae bacterium), alongside EcoRI and XbaI restriction enzymes, were procured from New England Biolabs (Ipswich, MA, USA). For the construction of a positive control vector, the N coding sequence of the aMPV/C reference strain (GenBank: AM293284.1) was chemically synthesized and subcloned into the pcDNA3.1 vector featuring a Flag-tag. All oligonucleotides were commissioned to EZassay Ltd. for commercial synthesis and HPLC-grade purification.

### 2.3. In Silico Design of Primers and CRISPR RNAs

The conservation of the RPA primer and crRNA target regions, including the TTTV PAM site (nucleotides 917–919 of the N gene), was confirmed by in silico analysis of all available aMPV/C N-gene sequences from GenBank using ClustalW 2.1 and ESPript 3.2 (see Supplementary Material). Bioinformatic screening for specific RPA primer pairs targeting the *N* gene of aMPV/C was conducted via the proprietary web-based platform https://www.ezassay.com/primer (accessed on 18 March 2025). Four candidate pairs were computationally generated, adhering to constraints of 150–250 bp for amplicon size and 25–35 nucleotides for primer length. In tandem, crRNA architectures were engineered to recognize loci immediately upstream of the 5′-TTTV-3′ protospacer adjacent motif (PAM). Each synthetic crRNA molecule was assembled with a 5′-T7 promoter sequence (UAAUACGACUCACUAUA), the canonical Cas12a scaffold (UAAUUUCUACUAAGUGUAGAU), and a 20–25 nt variable spacer complementary to the target genomic region. The crRNA Design interface (https://www.ezassay.com/rna, accessed on 7 July 2025) facilitated sequence optimization through predictive scoring algorithms. Comprehensive sequence data are tabulated in Table 1.

### 2.4. Integrated Isothermal Amplification and CRISPR/Cas12a Cleavage

RT-RPA reactions were performed in strict accordance with the manufacturer’s technical manual for the one-step RT-RPA kit. Briefly, a 20 μL master mix was prepared containing 10.0 μL of RT-RPA rehydration buffer (pre-formulated with reverse transcriptase to support integrated reverse transcription and isothermal amplification), 8.0 μL of nuclease-free water, and 0.5 μL each of forward and reverse primers (20 μM). Template DNA (1 μL) was introduced to initiate polymerization. Following thorough vortexing and centrifugation, the mixtures were incubated at 37 °C for 20 min within a constant-temperature metal bath supplied by Gingko Biotech (Beijing, China).

Subsequent collateral cleavage assessment was performed in a 20 μL Cas12a system comprising 5 μL of the RPA amplicon, 1 μL of LbCas12a protein, 2 μL of 10× Cas12a reaction buffer, 1 μL of the refined crRNA, and 0.6 μL of a fluorophore-quencher modified ssDNA reporter (FAM-TTATT-BHQ, 4 μM), and 10.4 μL of nuclease-free water (ddH_2_O). The enzymatic reaction was sustained at 37 °C for 20 min. Fluorescence intensity was quantitatively measured using a Tecan Infinite M200 microplate reader (Männedorf, Switzerland) configured to excitation and emission wavelengths of 492 nm and 521 nm, respectively. Gel documentation was performed using imaging equipment manufactured by Thermo Fisher Scientific (Waltham, MA, USA) and sourced from Gene Company Limited (Chai Wan, Hong Kong).

### 2.5. Lateral Flow Chromatography for Visual Signal Transduction

For point-of-care interpretation, 2 μL of the post-cleavage reaction product was admixed with 78 μL of dedicated running buffer. The resulting solution was immediately loaded onto the sample pad of the LFD. Chromatographic migration was permitted for 2 min at ambient temperature. Result adjudication relied on the following principle: the appearance of a single burgundy band exclusively at the control line (C) indicated a functional test device and a negative result, whereas the concurrent emergence of bands at both the control (C) and test (T) lines signified a positive outcome.

### 2.6. Dose–Response Profiling of CRISPR Effector Complexes

A systematic titration of Cas12a protein and crRNA concentrations was executed to maximize the signal-to-noise ratio. Gradients spanning Cas12a (10, 20, 30, 40, and 50 nmol/L) and crRNA (5, 10, 20, 30, 40 and 50 nmol/L) were empirically evaluated. Quantitative analysis of endpoint fluorescence identified 30 nmol/L Cas12a paired with 40 nmol/L crRNA as the stoichiometrically optimal formulation for subsequent diagnostic validation.

### 2.7. Determination of Analytical Sensitivity, Specificity and Repeatability

The limit of detection (LOD) was empirically defined by assaying 10-fold serial dilutions of the pcDNA3.1-aMPV/C N-Flag plasmid standard, with nuclease-free water serving as the template-free negative control. To ascertain diagnostic specificity, the assay was rigorously challenged against nucleic acid extracts derived from seven taxonomically distinct avian pathogens: *Mycoplasma anatis* (Ma, Strain:FJ2065), Duck plague virus (DPV, Strain: FJ1501), Duck tembusu virus (DTMUV, Strain: FJMH220, GenBank:JQ928189.1) and Duck reovirus (DRV, Strain: LH01), Avian Influenza virus (AIV, Strain: FJMH220), Muscovy Duck Parvovirus (MDPV, Strain: FJM5, GenBank: KR075689.1), Duck hepatitis virus (DHV, Strain: HB, GenBank: PP713235).

Repeatability of the RT-RPA-CRISPR/Cas12a-LFD assay was validated using two aMPV/C RNA standards (1.3 × 10^4^ and 1.3 × 10^2^ copies/µL) with three replicates each; nuclease-free ddH_2_O served as the negative control. Intra-assay consistency was evaluated across replicates within a single run, and inter-assay consistency across three independent runs on separate days. Reproducibility was defined by 100% concordance of LFD readouts across all tests.

### 2.8. Conventional qPCR for Comparative Analysis

A pair of specific primers targeting the conserved region of the aMPV/C *N* gene was designed: aMPV/C-F (5′-ACGAGAAAAAAACGCAT-3′) and aMPV/C-R (5′-AGAGACATTTTTACTTGTCCC-3′). The 20 μL reaction mixture consisted of 10 μL 2 × qPCR Master Mix, 0.5 μL of each forward and reverse primer (20 μM), 2 μL template, and nuclease-free ddH_2_O to adjust the final volume. Thermal cycling conditions were as follows: initial denaturation at 95 °C for 2 min, followed by 35 cycles of 95 °C for 15 s and 60 °C for 60 s.

### 2.9. Statistical Analysis

Data curation and graphical rendering were executed using GraphPad Prism 9 (GraphPad Software, Inc., Boston, MA, USA). Inter-group statistical significance was computed via two-tailed Student’s *t*-tests. All quantitative metrics are expressed as the arithmetic mean ± standard error of the mean (SEM) derived from three independent biological replicates. A probability (*p*) value inferior to 0.05 was predefined as the threshold for statistical significance. Probit analyses were conducted at the 95% confidence level. Cohen’s Kappa coefficients and their 95% confidence intervals were calculated to evaluate the diagnostic agreement between RT-RPA-CRISPR/Cas12a-LFD results and the reference qPCR assay.

The intensity of the test line (T) on each LFD strip was quantified via grayscale analysis using ImageJ 1.54p software (National Institutes of Health, Bethesda, MD, USA). A specimen was categorized as positive when the grayscale value exceeded the mathematically defined threshold: Threshold = Mean (Grayscale_negative_) + 3× SD(Grayscale_negative_), based on the pixel intensity values of known-negative clinical samples. Specimens with grayscale values below this threshold were recorded as negative.

## 3. Results

### 3.1. Schematic Illustration of the Detection Principle and Workflow

**Figure 1:**
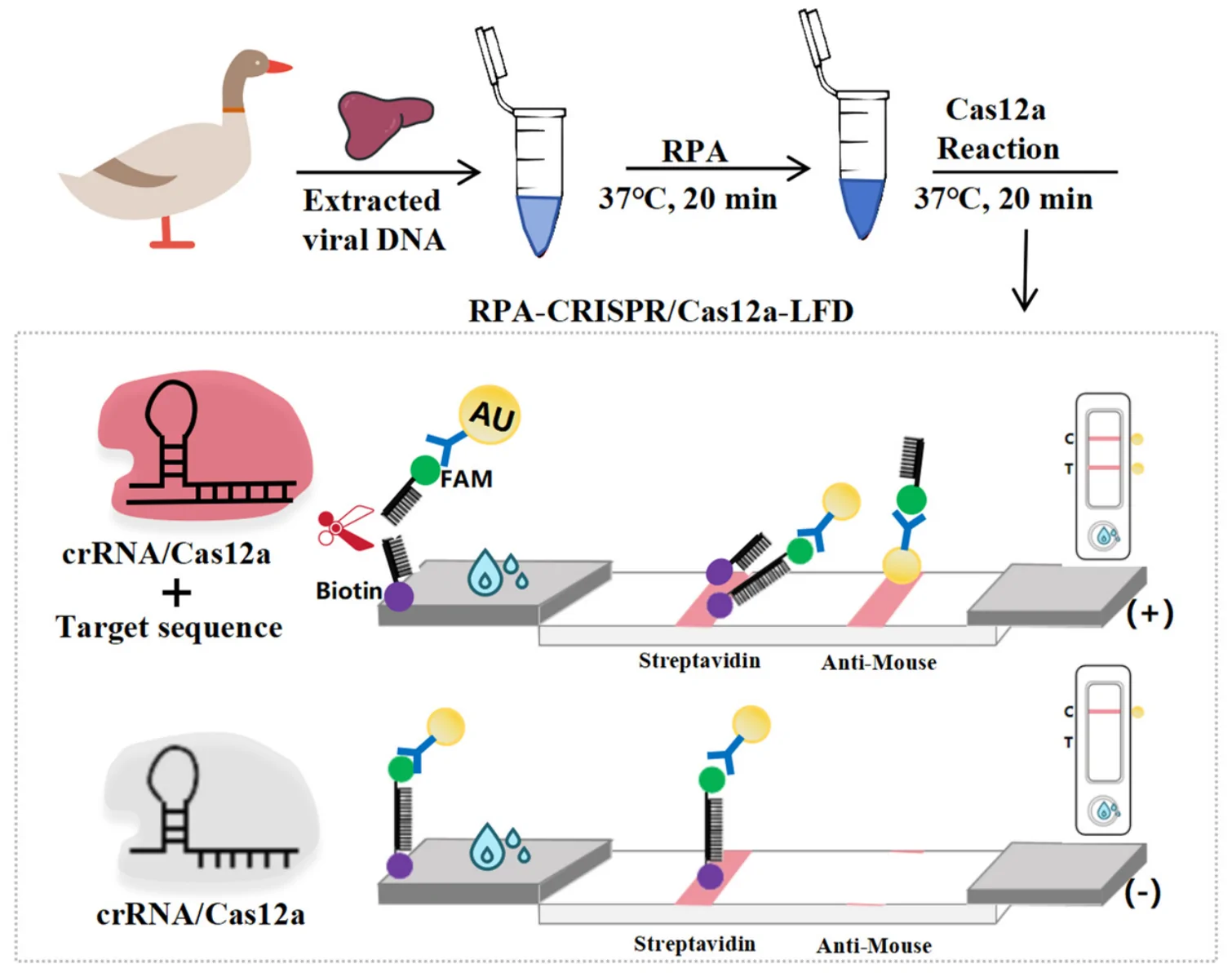
Schematic of the CRISPR/Cas12a diagnostic platform for aMPV/C detection. The workflow includes nucleic acid extraction, RT-RPA at 37 °C, CRISPR/Cas12a cleavage, and visual result interpretation via lateral flow dipstick (LFD). In the absence of target amplicons, the Cas12a protein remains inactive, and only the control line (C) appears. Upon target recognition, Cas12a is activated and cleaves the reporter, leading to the appearance of the test line (T) in addition to the control line.

The RT-RPA-CRISPR/Cas12a-LFD assay was designed to enable rapid, equipment-minimized detection of aMPV/C. As illustrated in Figure 1, the experimental workflow consists of four steps. First, nucleic acids are extracted from the lysates of various clinical samples, including oropharyngeal swabs, cloacal swabs, and tissue specimens (lung, trachea, and oviduct). Second, the extracted nucleic acids are amplified via RT-RPA in a 37 °C metal bath for 20 min to ensure adequate target amplification. Third, the CRISPR/Cas12a system is employed to detect the RT-RPA amplicons. Finally, results are interpreted using lateral flow strips. The underlying detection mechanism is based on Cas enzyme activation: without amplicons, the Cas protein remains inactive, and the intact reporter directs all gold nanoparticles (GNPs) to the control line, leaving the test line uncolored. Conversely, when the target nucleic acid is present and the Cas protein is activated, the reporter is cleaved. The GNPs bound to the cleaved fragments are captured at the test line, forming a visible colored band. Because the probe in the sample pad is in excess, both the control and test lines show color in a positive result.

### 3.2. Optimization of Reaction Parameters

**Figure 2:**
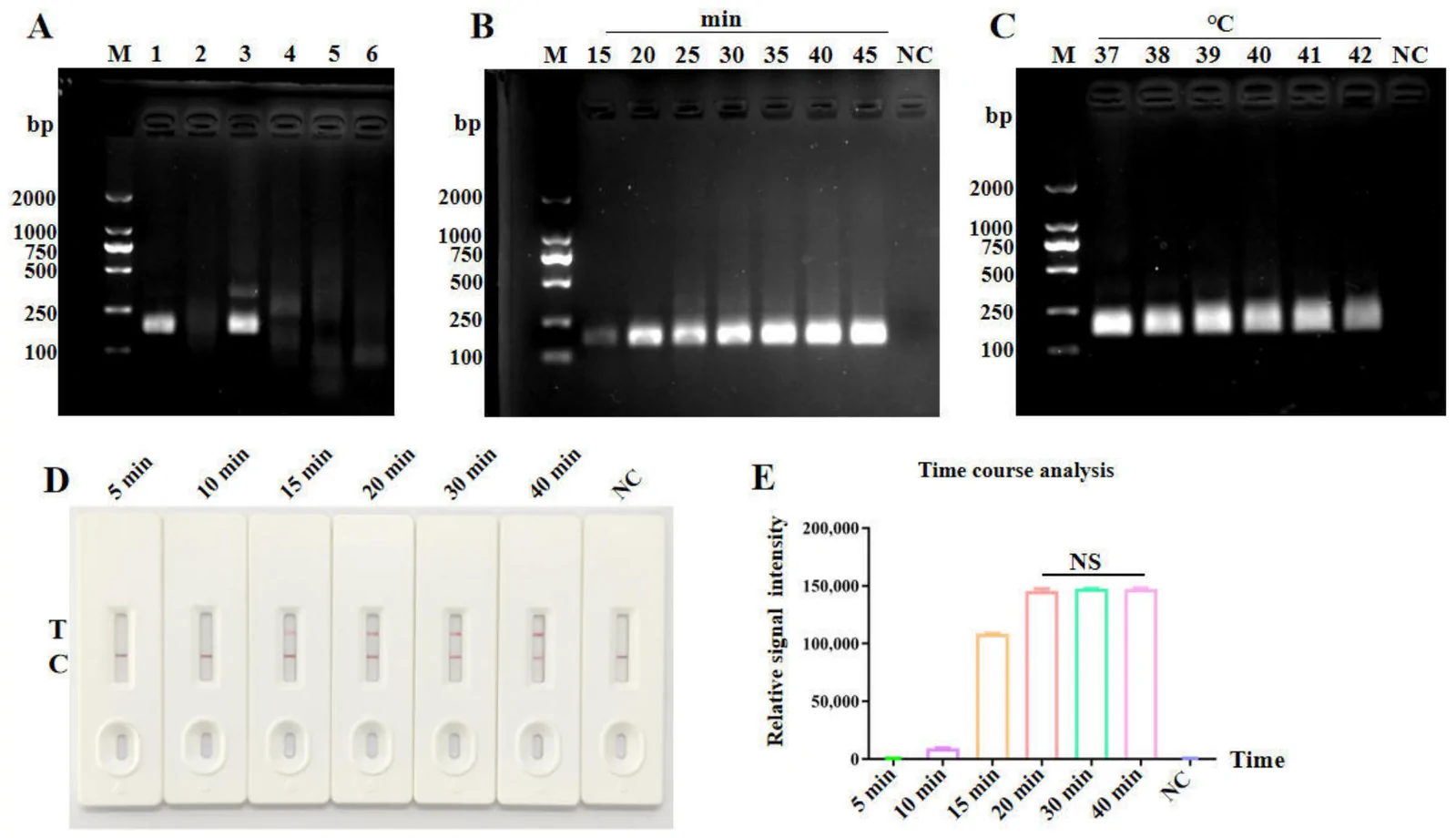
Optimization of Reaction Parameters. (**A**) Screening of RPA primer pairs by 1% agarose gel electrophoresis. M: DL2000 DNA Marker. Lanes 1–6: Amplification products of primer pairs F1-2/R1, F1-2/R1-H_2_O (negative control), F1-2/R2, F1-2/R2-H_2_O (negative control), F3/R3, and F3/R3-H_2_O (negative control). (**B**,**C**) Optimization of RPA reaction temperature and duration. (**D**) LFD results showing the effect of varying CRISPR/Cas12a reaction times. (**E**) Quantitative analysis of LFD test-line band intensities corresponding to different reaction times. NS indicates no significant difference.

Rational primer design is critical to RPA efficiency. Initial screening of three primer pairs targeting the *N* gene was performed via agarose gel electrophoresis. While the first two pairs successfully amplified the expected 220 bp products, primer pair #1 yielded the cleanest and most specific band (Figure 2A). We subsequently optimized the RPA reaction temperature and duration. As shown in Figure 2B,C, incubation at 37 °C for 20 min produced a distinct and intense band. Furthermore, we evaluated the optimal incubation time for the CRISPR/Cas12a reaction. Figure 2D,E demonstrate that clear positive bands were visible after 15 min, with no statistically significant difference in band intensity observed between 20 and 40 min. Consequently, primer pair #1 coupled with RPA at 37 °C for 20 min was established as the optimal amplification condition. Additionally, a 20 min CRISPR/Cas12a reaction was deemed sufficient for reliable signal detection. These standardized parameters were employed for all subsequent experiments.

### 3.3. Optimization of Cas12a and crRNA Concentrations

**Figure 3:**
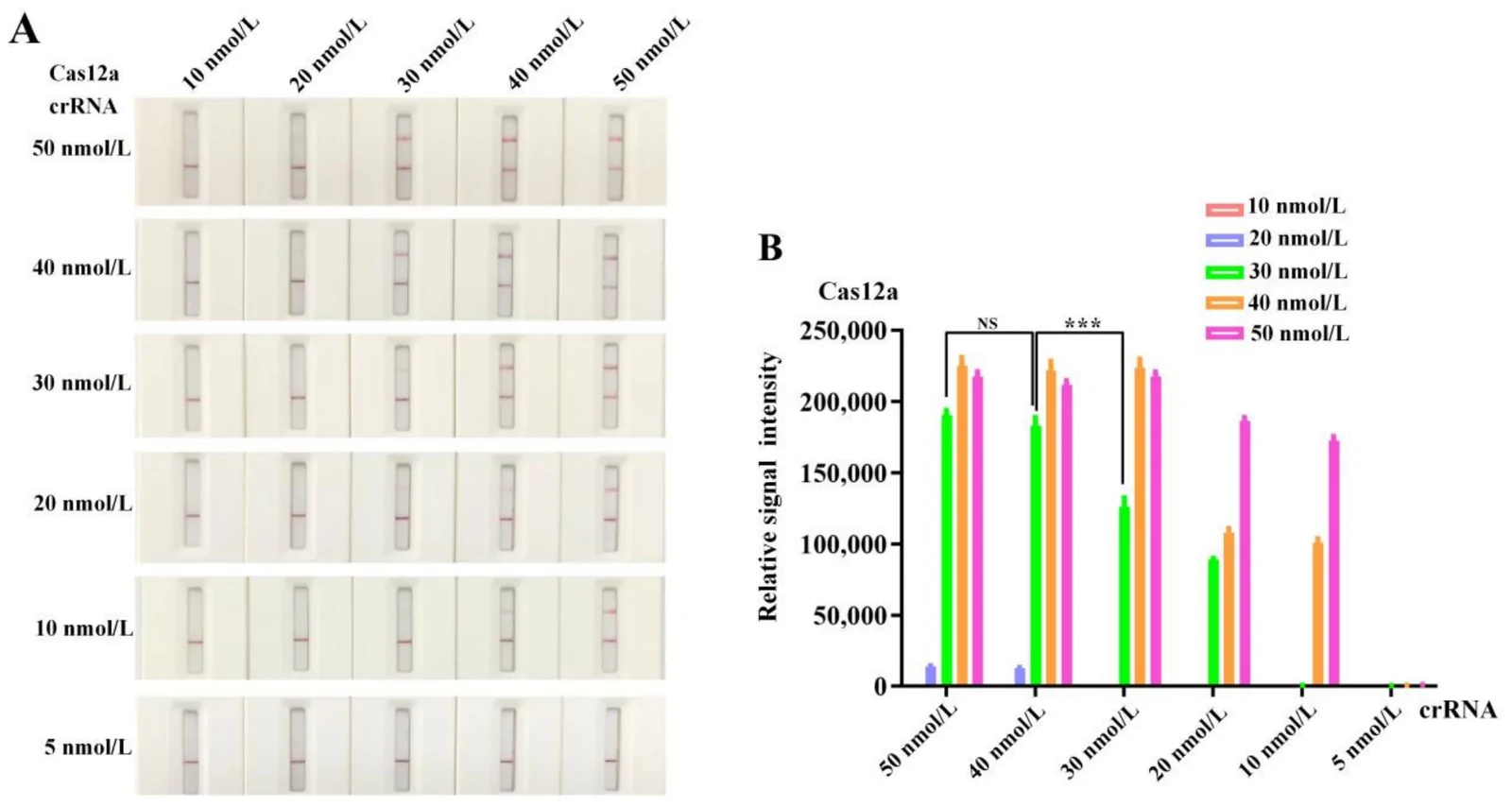
Optimization of the RPA and CRISPR/Cas12a system. (**A**) Fluorescence intensity profiles of the CRISPR/Cas12a reaction with gradient concentrations of Cas12a and crRNA. (**B**) Quantification of test-line band intensities from LFDs using ImageJ 1.54p software. Asterisks indicate significant differences (*** *p* < 0.001); NS indicates no significant difference.

To determine the optimal stoichiometry of the CRISPR effector complex, gradient concentrations of Cas12a (10–50 nmol/L) and crRNA (5–50 nmol/L) were systematically evaluated. As shown in Figure 3A,B, a distinct band at the test line became visible only when the Cas12a concentration reached 30 nmol/L. Within this fixed Cas12a concentration, elevating the crRNA concentration from 30 nmol/L to 40 nmol/L resulted in a significant increase in band intensity. However, further increasing the crRNA concentration to 50 nmol/L yielded no statistically significant enhancement in signal strength. Therefore, to balance reagent cost with detection performance, the combination of 30 nmol/L Cas12a and 40 nmol/L crRNA was established as the optimal formulation for subsequent experiments.

### 3.4. Analytical Sensitivity Assessment

**Figure 4:**
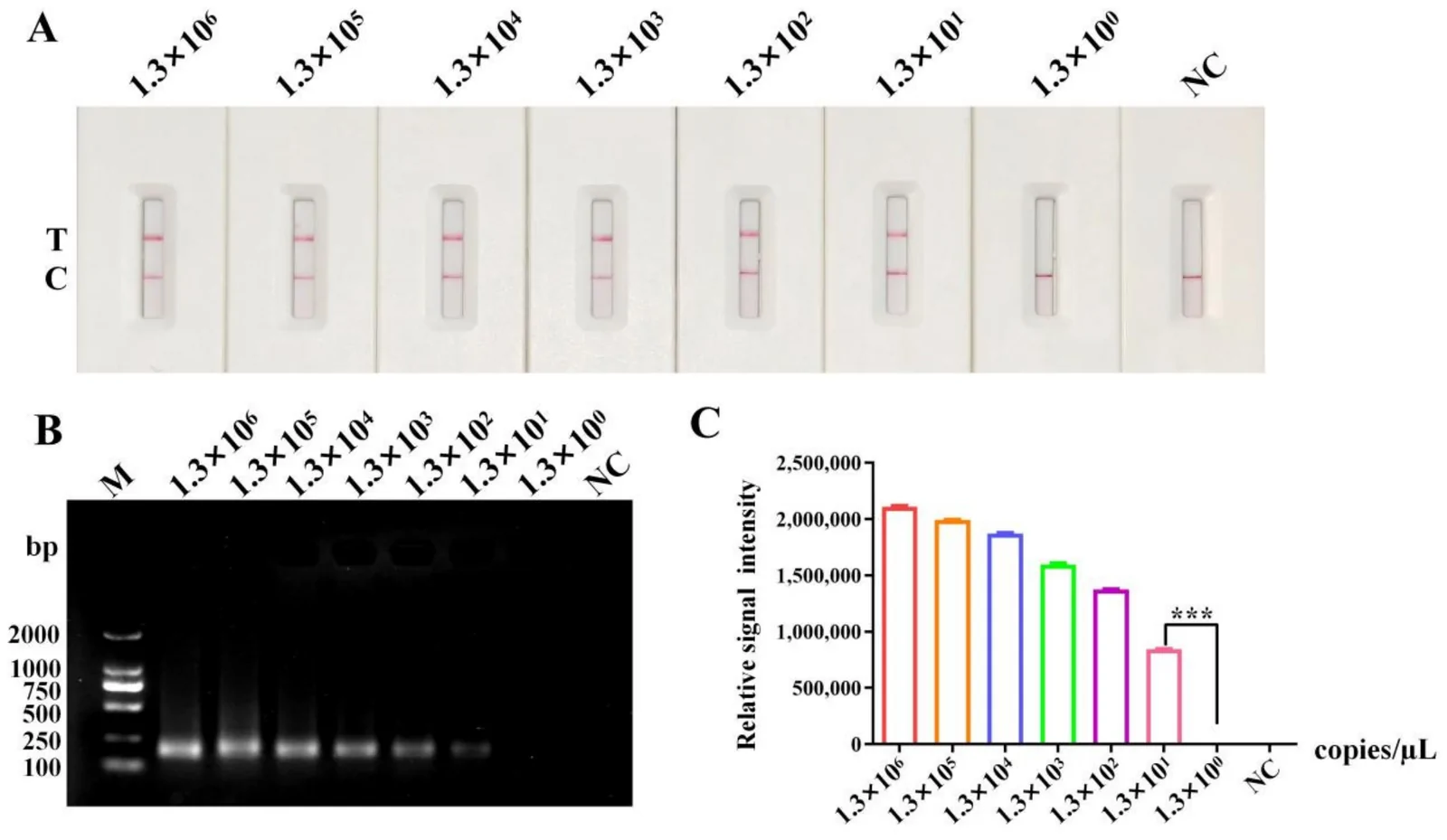
Sensitivity analysis. (**A**) LFD results showing detection sensitivity across a 10-fold serial dilution series of the plasmid standard (1.3 × 10^6^ to 1.3 × 10^0^ copies/µL). NC: Negative control. (**B**) Verification of RPA amplicons by agarose gel electrophoresis. (**C**) Quantitative analysis of test-line band intensities from the LFD strips. Asterisks indicate significant differences (*** *p* < 0.001).

The LOD was determined using 10-fold serial dilutions of the pcDNA3.1-aMPV/C N-Flag plasmid standard, spanning a concentration range of 1.3 × 10^6^ to 1.3 × 10^0^ copies/μL. Following RPA, CRISPR/Cas12a-LFD analysis identified the detection threshold at 1.3 × 10^1^ copies/μL: a distinct band was visible on the LFD test line at this dilution (Figure 4A), which was further corroborated by the presence of the expected 220 bp amplicon in agarose gel electrophoresis of the corresponding RPA product (Figure 4B). Quantitative analysis of LFD test-line band intensities (Figure 4C) revealed signals at 1.3 × 10^1^ copies/µL were significantly higher than those at 1.3 × 10^0^ copies/µL. Taken together, the LOD of the established assay was definitively set at 1.3 × 10^1^ copies/μL.

### 3.5. Analytical Specificity Evaluation

**Figure 5:**
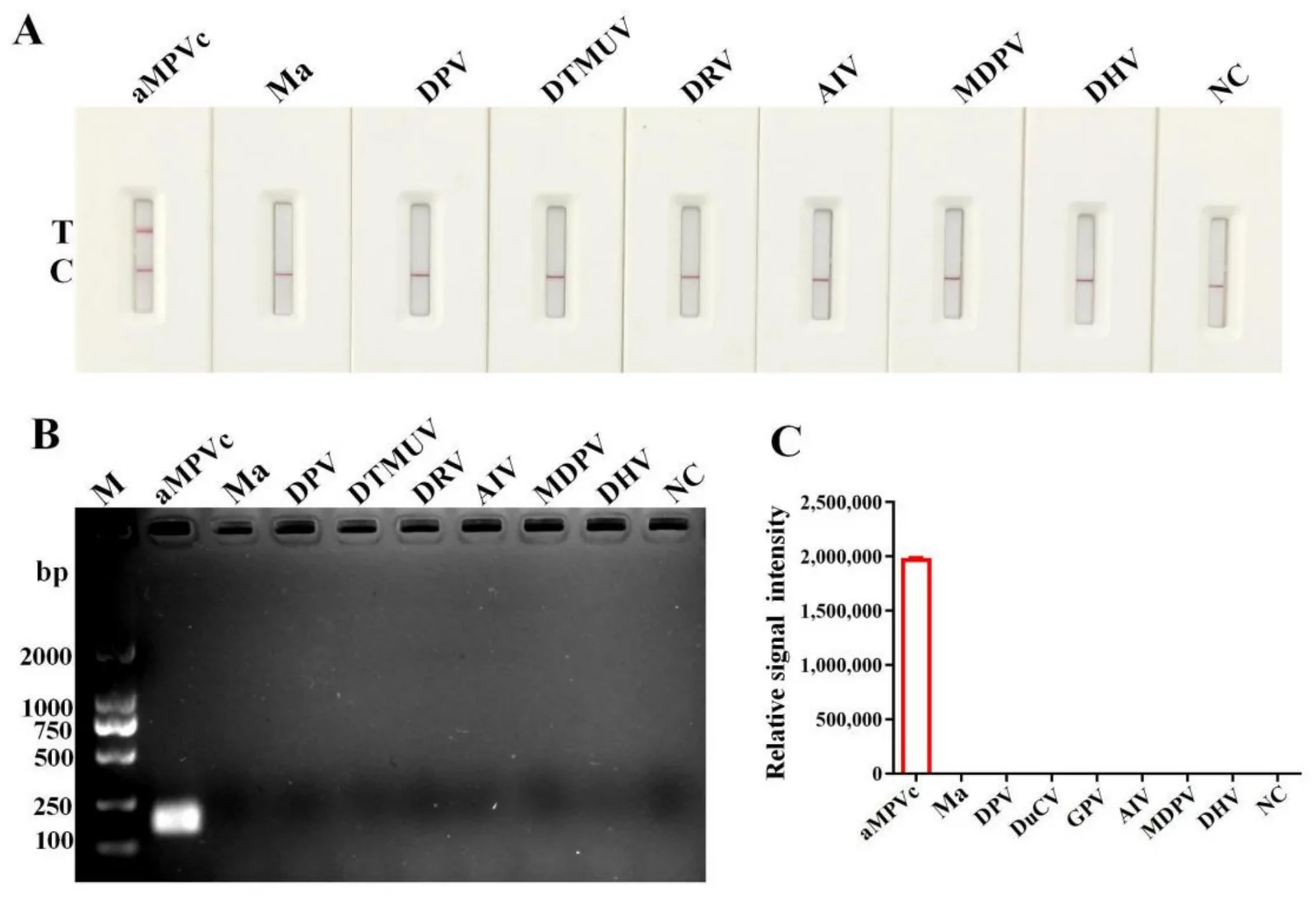
Specificity analysis. Genomic nucleic acids extracted from seven common non-target avian pathogens (Ma, DPV, DTMUV, DRV, AIV, MDPV, and DHV) were used as templates for the RPA-CRISPR/Cas12a-LFD assay. (**A**) LFD readouts. A positive signal (test line, T) is observed exclusively with aMPV/C. (**B**) Agarose gel electrophoresis of RPA amplicons confirms amplification only in the aMPV/C lane. (**C**) Quantitative analysis of LFD test-line band intensities. NC: no-template negative control.

The specificity of the established assay was rigorously validated against nucleic acids extracted from seven non-target avian pathogens (Ma, DPV, DTMUV, DRV, AIV, MDPV, DHV). As demonstrated by LFD readouts (Figure 5A), agarose gel electrophoresis of RPA amplicons (Figure 5B) and quantitative analysis of band intensities (Figure 5C), a distinct signal was exclusively detected in the presence of aMPV/C nucleic acids. No cross-reactivity was observed with any of the heterologous pathogens, confirming the high specificity of the method.

### 3.6. Assay Reproducibility

**Figure 6:**
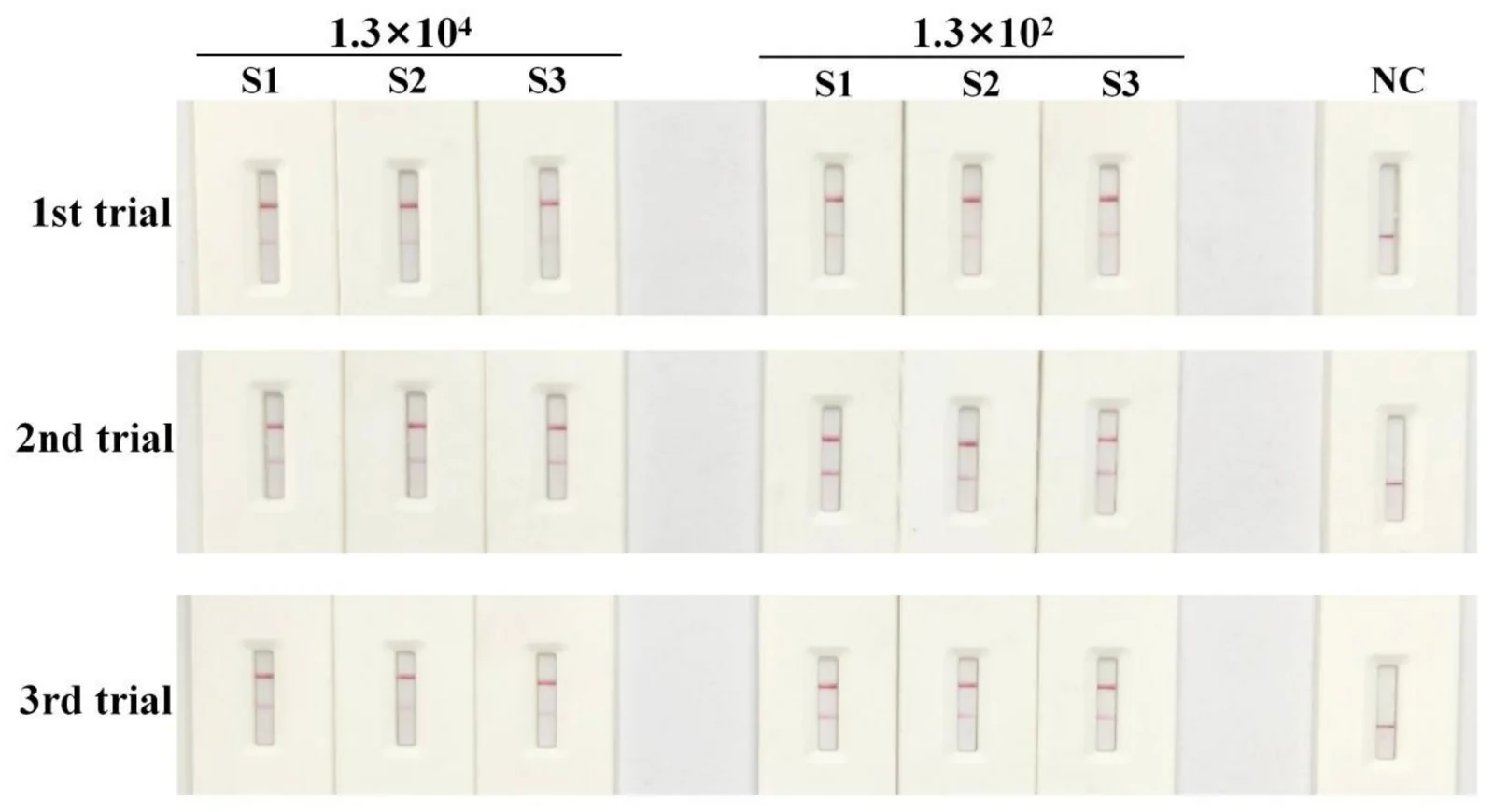
Reproducibility analysis. Representative LFD results for high (1.3 × 10^4^ copies/µL) and low (1.3 × 10^2^ copies/µL) concentration standards across three independent trials. Each trial included three replicates per concentration. Complete concordance of T-line signals was observed among replicates, with no signal detected in the negative controls.

The repeatability of the assay was assessed using high (1.3 × 10^4^copies/µL) and low (1.3 × 10^2^ copies/µL) concentration standards, with three independent runs and three LFD replicates per run. Qualitative observation of the LFDs (Figure 6) revealed clear and consistent test T line development across all replicates for both concentrations, while the NC lanes remained blank. These findings confirm the high precision and stability of the RT-RPA-CRISPR/Cas12a-LFD platform.

### 3.7. Clinical Sample Validation

**Figure 7:**
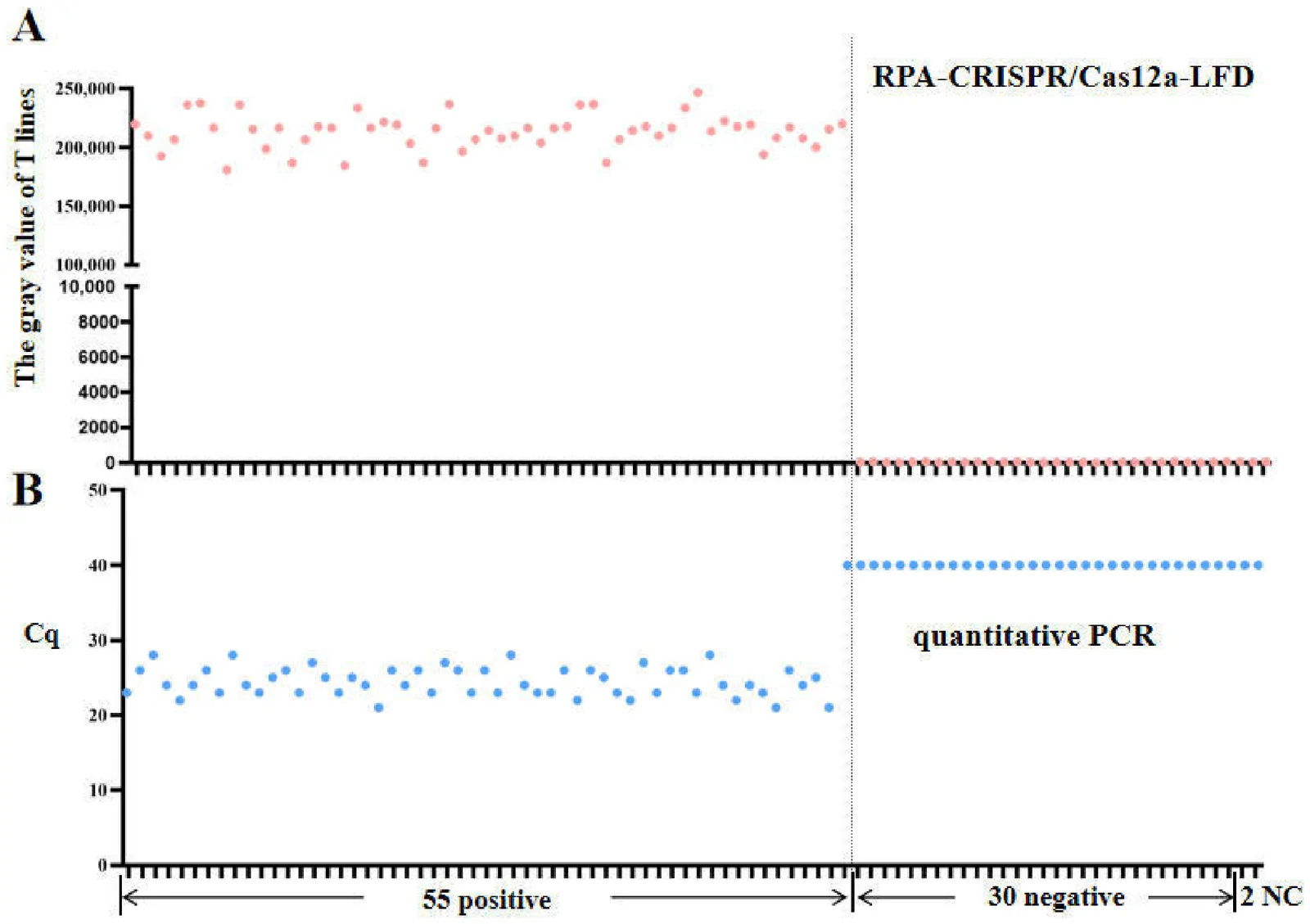
Clinical performance evaluation of the RPA-CRISPR/Cas12a-LFD assay. (**A**) Grayscale intensities of the LFD test lines (T) were quantified using ImageJ software, with a visible test-line band defined as a positive result. (**B**) Matched qPCR results for the same 87 clinical specimens. NC: Negative control.

The clinical applicability of the RT-RPA-CRISPR/Cas12a-LFD assay was validated using 87 field-collected duck samples. The intensity of the test line (T) on the LFD was quantified via grayscale analysis using ImageJ software (National Institutes of Health, Bethesda, MD, USA). Based on established grayscale thresholds, specimens were categorized into 55 positive and 30 negative cases (Figure 7). Comparative analysis against our previously established qPCR assay yielded a concordance rate of 98.85% (Table 2), underscoring the diagnostic reliability of this method for detecting aMPV/C in clinical settings.

## 4. Discussion

aMPV poses a persistent and escalating threat to global poultry health, a challenge particularly acute in the densely populated poultry sectors of Asia. As outlined in the Introduction, the taxonomic complexity of this pathogen-spanning subtypes A through D-coupled with the widespread prevalence of subtype C in China [1,2,3], underscores the urgent need for field-deployable surveillance tools. The economic ramifications are severe: beyond direct mortality, the virus induces significant production losses through egg-drop syndromes and secondary bacterial complications. Consequently, there is a critical demand for a diagnostic tool that not only exhibits high accuracy but is also operable at the farm level to interrupt these cascading adverse effects.

Traditional diagnostic modalities for aMPV/C remain misaligned with field exigencies. Viral isolation, while specific, is protracted (48–72 h), rendering it ineffective for informing immediate biosecurity decisions. Serological assays, reliant on antibody detection, are inherently retrospective and cannot distinguish between active viral shedding and historical exposure-a critical shortcoming during outbreak containment. Although conventional PCR offers high sensitivity, its reliance on thermal cyclers and specialized laboratory infrastructure creates a diagnostic void in resource-limited or remote farming communities. Alternative isothermal techniques, such as LAMP, have attempted to bridge this gap; however, their requirement for four to six primers and susceptibility to false positives due to primer-dimer formations compromise field reliability [15]. Similarly, standalone RPA, despite its rapidity, suffers from specificity deficits where non-specific amplification can lead to erroneous clinical interpretations [16,17].

To address these longstanding limitations, our study integrates the speed of RPA with the sequence-stringent collateral cleavage activity of CRISPR/Cas12a. By targeting a highly conserved yet specific region within the *N* gene, we ensured robust reactivity against circulating aMPV/C variants while guaranteeing absolute specificity. Notably, we identified an optimal formulation of 30 nmol/L Cas12a paired with 40 nmol/L crRNA. The assay achieved a LOD of 1.3 × 10^1^copies/μL, outperforming conventional PCR and enabling the detection of low-level viral shedding during early or convalescent stages of infection. Furthermore, the system demonstrated absolute analytical specificity, exhibiting no cross-reactivity with seven common non-target avian pathogens, which attests to the high-fidelity target recognition conferred by the CRISPR component.

When contextualized within recent advancements in CRISPR-based detection, the unique merits of our platform become evident. While previous studies have reported MIRA-CRISPR/Cas13a systems for chicken-origin aMPV or ERA/RPA-CRISPR/Cas12a platforms for other avian pathogens (e.g., duck Tembusu virus, swine influenza) [25,26,27], these methodologies often rely on fluorescence readouts or complex temperature profiling. In contrast, our RPA-Cas12a-LFD assay employs a DNA-targeted mechanism at a constant 37 °C, circumventing the T7 transcription step required by Cas13a systems and thereby significantly reducing operational complexity. Crucially, the integration of the lateral flow dipstick (LFD) enables a purely visual readout, eliminating the need for expensive fluorescence readers and facilitating rapid on-site detection.

The true utility of this assay lies in its translational potential for clinical practice. Validation using 87 clinical field samples-collected from Muscovy ducks exhibiting typical clinical signs in Fujian Province-demonstrated a 98.85% concordance rate with qPCR, confirming its robustness in real-world deployment. Diverging from laboratory-bound assays, our method requires only a simple portable heating block and yields intuitive visual results within one hour of sample collection. The interpretation logic is straightforward: a single band at the control line (C) indicates proper device function and a negative result, whereas dual bands at the C and test (T) lines confirm target presence, minimizing subjective reading errors. Even at the LOD concentration (1.3 × 10^1^ copies/μL), the T line remained distinctly visible, effectively mitigating controversies regarding subjectivity often associated with faint LFD bands in weak-positive samples. It should be noted that clinical validation was performed exclusively using Muscovy duck samples. Although turkeys are the primary host for severe aMPV manifestations and aMPV/C was first isolated from turkeys, its host range has expanded to include various avian species. In China, aMPV/C predominantly causes respiratory disease and egg-drop in ducks, with seroprevalence reaching 86% in some regions. Given that this study aimed to develop a diagnostic tool for the duck industry, validation was intentionally focused on the target host. Future studies will assess the assay’s performance in turkeys and other avian species.

Although the RT-RPA-CRISPR/Cas12a-LFD platform offers significant advantages in terms of speed, sensitivity, and visual interpretation, it is important to acknowledge a current limitation: the nucleic acid extraction step still relies on a commercial kit and standard laboratory equipment and is not yet fully field-deployable. Thus, while the amplification and detection steps are truly equipment-minimized, the overall workflow is not yet completely instrument-free. In addition to the sample preparation limitation noted above, several limitations warrant acknowledgment. Liquid RPA reagents are susceptible to degradation under extreme field conditions characterized by high temperature and humidity. While our repeatability assays confirmed stability under controlled laboratory settings, field deployment necessitates the adoption of lyophilized (dry-powder) reagent formulations. Such formats can withstand transportation and storage in simple cool boxes without stringent cold-chain infrastructure, significantly enhancing field resilience. Additionally, although our low-concentration optimization has reduced reagent consumption, the current cost of commercial Cas12a enzymes and synthetic crRNAs remains a bottleneck for large-scale grassroots implementation. Nevertheless, we anticipate that ongoing maturation of the biotechnology sector will drive down these costs, facilitating broader adoption.

Looking ahead, we aim to expand the utility of this platform along three core trajectories. First, we will develop multiplex detection capabilities by designing orthogonal crRNAs and utilizing multi-line LFDs to enable simultaneous detection of aMPV and other prevalent duck respiratory pathogens [28], such as avian influenza virus and duck Tembusu virus. Second, we plan to develop a direct-from-sample workflow using crude swab lysates, thereby eliminating the need for commercial extraction kits and further enhancing the field applicability of the assay. Future studies will also address current formulation limitations and will include comparative evaluation of multiple isothermal platforms (including RT-LAMP-LFD) in complex clinical matrices under field conditions. Third, drawing inspiration from recent dual-mode CRISPR platforms for human metapneumovirus, we plan to integrate the LFD with smartphone-based readers. By coupling the strip with a portable optical adapter, qualitative visual bands can be converted into quantitative, GPS-tagged data uploadable to cloud-based epidemiological databases [29]. This integration will elevate field diagnostics from simple binary results to a comprehensive, traceable public health surveillance system.

In conclusion, the RT-RPA-CRISPR/Cas12a-LFD platform described herein offers a practical tool for aMPV/C control. It successfully reconciles the competing demands of high sensitivity, high specificity, and operational simplicity. By enabling on-site, real-time decision-making, this assay holds strong potential to revolutionize epidemiological surveillance and outbreak management of aMPV/C in both smallholder and industrial farming systems, ultimately safeguarding poultry productivity and supporting sustainable poultry health management initiatives.

## 5. Conclusions

In this study, we established and validated an RT-RPA-CRISPR/Cas12a-LFD platform for rapid detection of avian metapneumovirus subtype C (aMPV/C) in clinical Muscovy duck samples. Using qPCR as the gold standard, the assay achieved a diagnostic concordance rate of 98.85% (86/87) with excellent specificity. The entire process requires only a simple heating block and a lateral flow dipstick, delivering visual results within 1 h and offering a practical alternative to conventional laboratory methods. Although the current liquid formulation requires cold-chain storage and further evaluation in crude samples is needed, this work lays a solid foundation for the development of field-deployable diagnostics. Future studies will focus on developing lyophilized reagents and conducting comparative evaluations of multiple isothermal platforms under field conditions.

## Figures and Tables

**Table 1:** The sequence of RPA primers and crRNA.

Name	Sequence (5′-3′)	Position
aMPV/c-N-RPA-F1-2	GGCCATGTCTCTGTACAAGCGGAACTCAAAC	820–850
aMPV/c-N-RPA-F3	TAGAGAGATGGGCCCCGAGTCAGGTCTTCTC	876–906
aMPV/c-N-RPA-R1	AGGCATTCCCTAGCACCACACTTGCAAAATT	958–988
aMPV/c-N-RPA-R2	GAGGCATTCCCTAGCACCACACTTGCAAAAT	958–989
aMPV/c-N-RPA-R3	TTTATCTTGTTGCTTTCTTTTAGGCTTCTTG	1064–1094
aMPV/c-N-crRNA	UAAUUUCUACUAAGUGUAGAUCCUCAGGUGGAGAAGACCUGACU	893–915

**Table 2:** The performance of RT-RPA-CRISPR compared with qPCR.

		qPCR			CR	Kappa (κ)
		Positive	Negative	Total		
RT-RPA-CRISPR/ Cas12a-LFD	Positive	54	1	55	98.85%	0.976
	Negative	0	32	32		
	Total	54	33	87		

## Data Availability

All data generated and analyzed during this study are included in this published article.

## Supplementary Materials

The following supporting information can be downloaded at: https://www.mdpi.com/article/10.3390/ani16193154/s1, Figure S1: in silico conservation analysis.
