# Supplementary material for: Visual and Equipment-Minimized Detection of Avian Metapneumovirus Subtype C by RT-RPA-CRISPR/Cas12a Coupled with Lateral Flow Dipstick
Source: Animals (Basel). 2026 Oct 8;16(19):3154. doi: 10.3390/ani16193154 (PMC13663432; doi:10.3390/ani16193154)
Supplement: Supplementary file 1 [file animals-16-03154-s001.zip › animals-4586834-supplementary.pdf]

```
                     1        10        20        30        40        50        60
EF199771.1_54-1238   ATGTCTCTTCAGGGGATTCAGCTTAGTGACTTGTCCTATAAGCATGCAATCCTTAAAGAA
AY028558.1_14-1196   ATGTCTCTTCAGGGGATTCAGCTTAGTGACTTGTCCTATAAGCATGCAATCCTTAAAGAA
EF199772.1_54-1238   ATGTCTCTTCAGGGGATTCAGCTTAGTGACTTGTCCTATAAGCATGCAATCCTTAAAGAA
FJ977568.1_54-1238   ATGTCTCTTCAGGGGATTCAGCTTAGTGACTTGTCCTATAAGCATGCAATCCTTAAAGAA
AY028559.1_14-1196   ATGTCTCTTCAGGGGATTCAGCTTAGTGACTTGTCCTATAAGCATGCAATCCTTAAAGAA
AF368173.1_14-1196   ATGTCTCTTCAGGGGATTCAGCTTAGTGACTTGTCCTATAAGCATGCAATCCTTAAAGAA
AY028557.1_14-1195   ATGTCTCTTCAGGGGATTCAGCTTAGTGACTTGTCCTATAAGCATGCAATCCTTAAAGAA
AY590688.1_54-1238   ATGTCTCTTCAGGGGATTCAGCTTAGTGACTTGTCCTATAAGCATGCAATCCTTAAAGAA
AF176590.1_14-1198   ATGTCTCTTCAGGGGATTCAGCTTAGTGACTTGTCCTATAAGCATGCAATCCTTAAAGAA
AY579780.1_54-1238   ATGTCTCTTCAGGGGATTCAGCTTAGTGACTTGTCCTATAAGCATGCAATCCTTAAAGAA
AY028556.1_14-1196   ATGTCTCTTCAGGGGATTCAGCTTAGTGACTTGTCCTATAAGCATGCAATCCTTAAAGAA
AY028568.1_14-1196   ATGTCTCTTCAGGGGATTCAGCTTAGTGATTTGTCCTATAAGCATGCAATCCTTAAAGAA
AY028564.1_14-1196   ATGTCTCTTCAGGGGATTCAGCTTAGTGATTTGTCCTATAAGCATGCAATCCTTAAAGAA
AY028562.1_14-1196   ATGTCTCTTCAGGGGATTCAGCTTAGTGATTTGTCCTATAAGCATGCAATCCTTAAAGAA
AY028561.1_14-1196   ATGTCTCTTCAGGGGATTCAGCTTAGTGATTTGTCCTATAAGCATGCAATCCTTAAAGAA
AY028565.1_14-1196   ATGTCTCTTCAGGGGATTCAGCTTAGTGATTTGTCCTATAAGCATGCAATCCTTAAAGAA
AY028563.1_14-1196   ATGTCTCTTCAGGGGATTCAGCTTAGTGATTTGTCCTATAAGCATGCAATCCTTAAAGAA
AY028567.1_14-1196   ATGTCTCTTCAGGGGATTCAGCTTAGTGATTTGTCCTATAAGCATGCAATCCTTAAAGAA
AY028560.1_14-1196   ATGTCTCTTCAGGGGATTCAGCTTAGTGATTTGTCCTATAAGCATGCAATCCTTAAGGAA
DQ009484.1_14-1198   ATGTCTCTTCAGGGGATTCAGCTTAGTGATTTGTCCTATAAGCATGCAATCCTTAAAGAA
AM293284.1_1-1185    ATGTCTCTTCAGGGGATTCAGCTTAGCGATTTGTCCTATAAGCATGCAATCCTTAAAGAA
HG934338.1_55-1239   ATGTCTCTTCAGGGGATTCAGCTTAGCGATTTGTCCTATAAGCATGCAATCCTTAAAGAA
PP662686.1_54-1238   ATGTCTCTTCAGGGGATTCAGCTTAGCGATTTGTCCTACAAGCATGCAATCCTTAAAGAA
OM179883.1_52-1236   ATGTCTCTTCAAGGGATTCAGCTTAGTGACTTGTCCTATAAGCATGCAATCCTTAAAGAA
OM256458.1_54-1238   ATGTCTCTTCAAGGGATTCAGCTTAGTGACTTGTCCTATAAGCATGCAATCCTTAAAGAA
PQ530038.1_1-1185    ATGTCTCTTCAGGGGATTCAGCTCAGTGACTTGTCCTATAAGCATGCAATCCTTAAAGAA
MZ274340.1_1-1185    ATGTCTCTTCAGGGGATTCAGCTCAGTGACTTGTCCTATAAGCATGCAATCCTTAAAGAA
KF364615.2_14-1198   ATGTCTCTTCAGGGGATTCAGCTTAGTGACTTGTCCTATAAGCATGCAATCCTTAAAGAA
MZ274341.1_1-1185    ATGTCTCTTCAGGGGATTCAGCTTAGTGACTCGTCCTATAAGCATGCAATCCTTAAAGAA
KC915036.1_54-1238   ATGTCTCTTCAGGGGATTCAGCTCAGTGACTTGTCCTATAAGCATGCAATCCTTAAAGAA
OR365552.1_54-1238   ATGTCTCTTCAGGGGATTCAGCTTAGTGACTTGTCCTATAAGCATGCAATCCTTAAAGAA
OR365551.1_54-1238   ATGTCTCTTCAGGGGATTCAGCTTAGTGACTTGTCCTATAAGCATGCAATCCTTAAAGAA
```

```
                              70        80        90       100       110       120
EF199771.1_54-1238   TCACAGTACACAATCAAAAGAGATGTGGGGACAACCACAGCTGTCACTCCATCTTCTCTG
AY028558.1_14-1196   TCACAGTACACAATCAAAAGAGATGTGGGGACAACCACAGCTGTCACTCCATCTTCTCTG
EF199772.1_54-1238   TCACAGTACACAATCAAAAGAGATGTGGGGACAACCACAGCTGTCACTCCATCTTCTCTG
FJ977568.1_54-1238   TCACAGTACACAATCAAAAGAGATGTGGGGACAACCACAGCTGTCACTCCATCTTCTCTG
AY028559.1_14-1196   TCACAGTACACAATCAAAAGAGATGTGGGGACAACCACAGCTGTCACTCCATCTTCTCTG
AF368173.1_14-1196   TCACAGTACACAATCAAAAGAGATGTGGGGACAACCACAGCTGTCACTCCATCTTCTCTG
AY028557.1_14-1195   TCACAGTACACAATCAAAAGAGATGTGGGGACAACCACAGCTGTCACTCCATCTTCTCTG
AY590688.1_54-1238   TCACAGTACACAATCAAAAGAGATGTGGGGACAACCACAGCTGTCACTCCGTCTTCTCTG
AF176590.1_14-1198   TCACAGTACACAATCAAAAGAGATGTGGGGACAACCACAGCTGTCACTCCGTCTTCTCTG
AY579780.1_54-1238   TCACAGTACACAATCAAAAGAGATGTGGGGACAACCACAGCTGTCACTCCATCTTCTCTG
AY028556.1_14-1196   TCACGGTACACAATCAAAAGAGATGTGGGGACAACCACAGCTGTCACTCCATCTTCTCTG
AY028568.1_14-1196   TCACAGTACACAATCAAAAGAGATGTGGGGACAACCACAGCTGTCACTCCATCTTCTCTG
AY028564.1_14-1196   TCACAGTACACAATCAAAAGAGATGTGGGGACAACCACAGCTGTCACTCCATCTTCTCTG
AY028562.1_14-1196   TCACAGTACACAATCAAAAGAGATGTGGGGACAACCACAGCTGTCACTCCATCTTCTCTG
AY028561.1_14-1196   TCACAGTACACAATCAAAAGAGATGTGGGGACAACCACAGCTGTCACTCCATCTTCTCTG
AY028565.1_14-1196   TCACAGTACACAATCAAAAGAGATGTGGGGACAACCACAGCTGTCACTCCATCTTCTCTG
AY028563.1_14-1196   TCACAGTACACAATCAAAAGAGATGTGGGGACAACCACAGCTGTCACTCCATCTTCTCTG
AY028567.1_14-1196   TCACAGTACACAATCAAAAGAGATGTGGGGACAACCACAGCTGTCACTCCTTCTTCTCTG
AY028560.1_14-1196   TCACAGTACACAATCAAAAGAGATGTGGGGACAACCACAGCTGTCACTCCATCTTCTCTG
DQ009484.1_14-1198   TCACAGTACACAATCAAAAGAGATGTGGGGACAACCCCAGCTGTCACTCCATCTTCTCTG
AM293284.1_1-1185    TCACAGTACACAATCAAAAGAGATGTGGGGACAACCACTGCTGTTACTCCATCTTCTTTG
HG934338.1_55-1239   TCACAGTACACAATCAAAAGAGATGTGGGGACAACCACTGCTGTTACTCCATCTTCTTTG
PP662686.1_54-1238   TCACAGTACACAATCAAAAGAGATGTGGGGACAACCACTGCTGTTACTCCATCTTCTCTG
OM179883.1_52-1236   TCACAGTACACAATAAAAAGAGATGTGGGGACAACCACTGCTGTTACTCCATCTTCTATG
OM256458.1_54-1238   TCACAGTACACAATAAAAAGAGATGTGGGGACAACCACTGCTGTTACTCCATCTCCTCTG
PQ530038.1_1-1185    TCACAGTACACAATAAAAAGAGATGTAGGGACAACCACTGCTGTTACTCCATCTTCTCTG
MZ274340.1_1-1185    TCACAGTACACAATAAAAAGAGATGTAGGGACAACCACTGCTGTTACTCCATCTTCTCTG
KF364615.2_14-1198   TCACAGTACACAATAAAAAGAGATGTAGGGACAACCACTGCTGTTACTCCATCTTCTCTG
MZ274341.1_1-1185    TCACAGTACACAATAAAAAGAGATGTAGGGACAACCACTGCTGTTACTCCATCTTCTCTG
KC915036.1_54-1238   TCACAGTACACAATAAAAAGAGATGTAGGGACAACCACTGCTGTTACTCCATCTTCTCTG
OR365552.1_54-1238   TCACAGTACACAATAAAAAGGGATGTGGGGACAACCACTGCTGTTACTCCATCTTCTCTG
OR365551.1_54-1238   TCACAGTACACAATAAAAAGGGATGTGGGGACAACCACTGCTGTTACTCCATCTTCTCTG
```

```
                          130       140       150       160       170       180
EF199771.1_54-1238   CAGAGGGAAGTGTCACTCTTATGTGGAGAGATACTGTATGCCAAGCACACAGATTACTCA
AY028558.1_14-1196   CAGAGGGAAGTGTCACTCTTATGTGGAGAGATACTGTATGCCAAGCACACAGATTACTCA
EF199772.1_54-1238   CAGAGGGAAGTGTCACTCTTATGTGGAGAGATACTGTATGCCAAGCACACAGATTACTCA
FJ977568.1_54-1238   CAGAGGGAAGTGTCACTCTTATGTGGAGAGATACTGTATGCCAAGCACACAGATTACTCA
AY028559.1_14-1196   CAGAGGGAAGTGTCACTCTTATGTGGAGAGATACTGTATGCCAAGCACACAGATTACTCA
AF368173.1_14-1196   CAGAGGGAAGTGTCACTCTTATGTGGAGAGATACTGTATGCCAAGCACACAGATTACTCA
AY028557.1_14-1195   CAGAGGGAAGTGTCACTCTTATGTGGAGAGATACTGTATGCCAAGCACACAGATTACTCA
AY590688.1_54-1238   CAGAGGGAAGTGTCACTCTTATGTGGAGAGATACTGTATGCCAAGCACACAGATTACTCA
AF176590.1_14-1198   CAGAGGGAAGTGTCACTCTTATGTGGAGAGATACTGTATGCCAAGCACACAGATTACTCA
AY579780.1_54-1238   CAGAGGGAAGTGTCACTCTTATGTGGAGAGATACTGTATGCCAAGCACACAGATTACTCA
AY028556.1_14-1196   CAGAGGGAAGTGTCACTCTTATGTGGAGAGATACTGTATGCCAAGCACACAGATTACTCA
AY028568.1_14-1196   CAGAGGGAAGTATCACTCTTATGTGGAGAGATACTGTATACCAAGCACACAGATTACTCA
AY028564.1_14-1196   CAGAGGGAAGTATCACTCTTATGTGGAGAGATACTGTATACCAAGCACACAGATTACTCA
AY028562.1_14-1196   CAGAGGGAAGTATCACTCTTATGTGGAGAGATACTGTATACCAAGCACACAGATTACTCA
AY028561.1_14-1196   CAGAGGGAAGTATCACTCTTATGTGGAGAGATACTGTATACCAAGCACACAGATTACTCA
AY028565.1_14-1196   CAGAGGGAAGTATCACTCTTATGTGGAGAGATACTGTATACCAAGCACACAGATTACTCA
AY028563.1_14-1196   CAGAGGGAAGTATCACTCTTATGTGGAGAGATACTGTATACCAAGCACACAGATTACTCA
AY028567.1_14-1196   CAGAGGGAAGTATCACTCTTATGTGGAGAGATACTGTATACCAAGCCCACAGATTACTCA
AY028560.1_14-1196   CAGAGGGAAGTATCACTCTTATGTGGAGAGATACTGTATACCAAGCACACAGATTACTCA
DQ009484.1_14-1198   CAGAGGGAAGTATCACTCTTATGTGGAGAGATACTGTATGCCAAGCACACAGATTACTCA
AM293284.1_1-1185    CAGAGGGAAGTATCACTTCTGTGCGGAGAGATACTGTATGCCAAGCACACAGATTACTCA
HG934338.1_55-1239   CAGAGGGAAATATCACTTCTGTGCGGAGAGATACTGTATGCCAAGCACACAGATTACTCA
PP662686.1_54-1238   CAGAGGGAAGTATCACTCCTATGTGGAGAGATATTGTATGCCAAGCACACAGATTACTCA
OM179883.1_52-1236   CAGAGGGAAGTATCACTCCTATGCGGAGAGATACTGTATGCCAAGCACACAGATTACTCA
OM256458.1_54-1238   CAGAGGGAAGTATCACTCCTATGCGGAGAGATACTGTATGCCAAACACACAGATTACTCA
PQ530038.1_1-1185    CAGAGGGAAGTATCACTCCTATGCGGAGAGATACTGTATGCCAAGCACACAGATTACTCA
MZ274340.1_1-1185    CAGAGGGAAGTATCACTCCTATGCGGAGAGATGCTGTATGCCAAGCACACAGATTACTCA
KF364615.2_14-1198   CAGAGGGAAGTATCACTCCTATGCGGAGAGATACTGTATGCCAAGCACACAGATTACTCA
MZ274341.1_1-1185    CAGAGGGAAGTATCACACCTATGCGGAGAGATACTGTATGCCAAGCACACAGATTACTCA
KC915036.1_54-1238   CAGAGGGAAGTATCACTCCTATGCGGAGAGATACTGTATGCCAAGCACACAGATTACTCA
OR365552.1_54-1238   CAGAGGGAAGTATCACTCCTATGCGGAGAGATACTGTATGCCAAGCACACAGATTACTCA
OR365551.1_54-1238   CAGAGGGAAGTATCACTCCTATGCGGAGAGATACTGTATGCCAAGCACACAGATTACTCA
```

```
                          190       200       210       220       230       240
EF199771.1_54-1238   CATGCAGCTGAAGTAGGAATGCAGTACGTGAGCACCACACTGGGAGCAGAGCGTACACAG
AY028558.1_14-1196   CATGCAGCTGAAGTAGGAATGCAGTACGTGAGCACCACACTGGGAGCAGAGCGTACACAG
EF199772.1_54-1238   CATGCAGCTGAAGTAGGAATGCAGTACGTGAGCACCACACTGGGAGCAGAGCGTACACAG
FJ977568.1_54-1238   CATGCAGCTGAAGTAGGAATGCAGTACGTGAGCACCACACTGGGAGCAGAGCGTACACAG
AY028559.1_14-1196   CATGCAGCTGAAGTAGGAATGCAGTACGTGAGCACCACACTGGGAGCAGAGCGTACACAG
AF368173.1_14-1196   CATGCAGCTGAAGTAGGAATGCAGTACGTGAGCACCACACTGGGAGCAGAGCGTACACAG
AY028557.1_14-1195   CATGCAGCTGAAGTAGGAATGCAGTACGTGAGCACCACACTGGGAGCAGAGCGTACACAG
AY590688.1_54-1238   CATGCAGCTGAAGTAGGAATGCAGTACGTGAGCACCACACTGGGAGCAGAGCGTACACAG
AF176590.1_14-1198   CATGCAGCTGAAGTAGGAATGCAGTACGTGAGCACCACACTGGGAGCAGAGCGTACACAG
AY579780.1_54-1238   CATGCAGCTGAAGTAGGAATGCAGTACGTGAGCACCACACTGGGAGCAGAGCGTACACAG
AY028556.1_14-1196   CATGCAGCTGAAGTAGGAATGCAGTACGTGAGCACCACACTGGGAGCAGAGCGTACACAG
AY028568.1_14-1196   CATACAGCTGAAGTAGGAATGCAGTATGTGAGCACCACACTGGGAGCAGAACGTACACAA
AY028564.1_14-1196   CATGCAGCTGAAGTAGGAATGCAGTGTGTGAGCACCACACTGGGAGCAGAACGTACACAA
AY028562.1_14-1196   CATGCAGCTGAAGTAGGAATGCAGTATGTGAGCACCACACTGGGAGCAGAACGTACACAA
AY028561.1_14-1196   CATGCAGCTGAAGTAGGAATGCAGTATGTGAGCACCACACTGGGAGCAGAACGTACACAA
AY028565.1_14-1196   CATGCAGCTGAAGTAGGAATGCAGTATGTGAGCACCACACTGGGAGCAGAACGTACACAA
AY028563.1_14-1196   CATGCAGCTGAAGTAGGAATGCAGTATGTGAGCACCACACTGGGAGCAGAACGTACACAA
AY028567.1_14-1196   CATGCAGCTGAAGTAGGAATGCAGTATGTGAGCACCACACTGGGAGCAGAACGTACACAA
AY028560.1_14-1196   CATGCAGCTGAAGTAGGAATGCAGTATGTGAGCACCACACTGGGAGCAGAACGTACACAA
DQ009484.1_14-1198   CATGCAGCTGAAGTAGGAATGCAGTATGTGAGCACCACACTGGGAGCAGAACGTACACAA
AM293284.1_1-1185    CATGCAGCAGAAGTAGGAATGCAGTATGTGAGTACCACACTGGGAGCAGAGCGTACACAG
HG934338.1_55-1239   CATGCAGCAGAAGTAGGAATGCAGTATGTGAGTACCACACTGGGAGCAGAGCGTACACAG
PP662686.1_54-1238   CATGCAGCAGAAGTAGGAATGCAGTACGTGAGCACCACATTAGGAGCAGAGCGTACACAG
OM179883.1_52-1236   CATGCAGCTGAAGTAGGAATGCAGTATGTAAGCACCACACTGGGAGCAGAGCGTACACAG
OM256458.1_54-1238   CATGCAGCTGAAGTAGGAATGCAGTATGTAAGCACCACACTGGGAGCAGAGCGTACACAG
PQ530038.1_1-1185    CATGCAGCTGAAGTAGGAATGCAGTACGTGAGCACCACATTGGGAGCAGAGCGTACACAG
MZ274340.1_1-1185    CATGCAGCTGAAGTAGGAATGCAGTGCGTGAGCACCACATTGGGAGCAGAGCGTACACAG
KF364615.2_14-1198   CATGCAGCTGAAGTAGGAATGCAGTACGTGAGCACCACATTGGGAGCAGAGCGTACACAG
MZ274341.1_1-1185    CATGCAGCTGAAGTAGGAATGCAGTACGTGAGCACCACATTGGGAGCAGGGCGTACACAG
KC915036.1_54-1238   CATGCAGCTGAAGTAGGAATGCAGTACGTGAGCACCACATTGGGAGCAGAGCGTACACAG
OR365552.1_54-1238   TATGCAGCTGAAGTAGGAATGCAGTATGTGAGCACCACACTGGGAGCAGAGCGCACACAG
OR365551.1_54-1238   TATGCAGCTGAAGTAGGAATGCAGTATGTGAGCACCACACTGGGAGCAGAGCGCACACAG
```

```
                           250       260       270       280       290       300
EF199771.1_54-1238  CAGATACTAAAGAACTCAGGTAGTGAGGTGCAGGCAGTATTGACCAAGACATACTCTCTT
AY028558.1_14-1196  CAGATACTAAAGAACTCAGGTAGTGAGGTGCAGGCAGTATTGACCAAGACATACTCTCTT
EF199772.1_54-1238  CAGATACTAAAGAACTCAGGTAGTGAGGTGCAGGCAGTATTGACCAAGACATACTCTCTT
FJ977568.1_54-1238  CAGATACTAAAGAACTCAGGTAGTGAGGTGCAGGCAGTATTGACCAAGACATACTCTCTT
AY028559.1_14-1196  CAGATACTAAAGAACTCAGGTAGTGAGGTGCAGGCAGTATTGACCAAGACATACTCTCTT
AF368173.1_14-1196  CAGATACTAAAGAACTCAGGTAGTGAGGTGCAGGCAGTATTGACCAAGACATACTCTCTT
AY028557.1_14-1195  CAGATACTAAAGAACTCAGGTAGTGAGGTGCAGGCAGTATTGACCAAGACATACTCTCTT
AY590688.1_54-1238  CAGATACTAAAGAACTCAGGTAGTGAGGTGCAGGCAGTATTGACCAAGACATACTCTCTT
AF176590.1_14-1198  CAGATACTAAAGAACTCAGGTAGTGAGGTGCAGGCAGTATTGACCAAGACATACTCTCTT
AY579780.1_54-1238  CAGATACTAAAGAACTCAGGTAGTGAGGTGCAGGCAGTATTGACCAAGACATACTCTCTT
AY028556.1_14-1196  CAGATACTAAAGAACTCAGGTAGTGAGGTGCAGGCAGTATTGACCAAGACATACTCTCTT
AY028568.1_14-1196  CAGATACTAAAGAACTCAGGTAGTGAGGTGCAGGCAGTGTTGACCAAGACATACTCTCTT
AY028564.1_14-1196  CAGATACTAAAGAACTCAGGTAGTGAGGTGCAGGCAGTGTTGACCAAGACATACTCTCTT
AY028562.1_14-1196  CAGATACTAAAGAACTCAGGTAGTGAGGTGCAGGCAGTGTTGACCAAGACATACTCTCTT
AY028561.1_14-1196  CAGATACTAAAGAACTCAGGTAGTGAGGTGCAGGCAGTGTTGACCAAGACATACTCTCTT
AY028565.1_14-1196  CAGATACTAAAGAACTCAGGTAGTGAGGTGCAGGCAGTGTTGACCAAGACATACTCTCTT
AY028563.1_14-1196  CAGATACTAAAGAACTCAGGTAGTGAGGTGCAGGCAGTGTTGACCAAGACATACTCTCTT
AY028567.1_14-1196  CAGATACTAAAGAACTCAGGTAGTGAGGTGCAGGCAGTGTTGACCAAGACATACTCTCTT
AY028560.1_14-1196  CAGATACTAAAGAACTCAGGTAGTGAGGTGCAGGCAGTGTTGACCAAGACATACTCTCTT
DQ009484.1_14-1198  CAGATACTAAAGAACTCAGGTAGTGAGGTGCAGGCAGTATTGACCAAGACATACTCTCTT
AM293284.1_1-1185   CAAATACTAAAGAACTCAGGTAGTGAGGTGCAAGCAGTATTGACCAAAACATACTCTCTT
HG934338.1_55-1239  CAGATACTAAAGAACTCAGGTAGTGAGGTGCAAGCAGTATTGACCAAAACATACTCTCTT
PP662686.1_54-1238  CAGATACTGAAGAACTCAGGTAGTGAGGTGCAAGCAGTGTTGACAAAAACATACTCTCTT
OM179883.1_52-1236  CAGATACTAAAGAACTCAGGTAGTGAGGTGCAGGCAGTATTGACCAAGACATACTCCCTT
OM256458.1_54-1238  CAGATACTAAAGAACTCAGGTAGTGAGGTGCAGGCAGTATTGACCAAGACATACTCCCTT
PQ530038.1_1-1185   CAGATACTAAAGAACTCAGGTAGTGAAGTGCAGGCAGTATTGACCAAGACATACTCCCTT
MZ274340.1_1-1185   CAGATACTAAAGAACTCAGGTAGTGAAGTGCAGGCAGTATTGACCAAGACATACTCCCTT
KF364615.2_14-1198  CAGATACTAAAGAACTCAGGTAGTGAAGTGCAGGCAGTATTGACCAAGACATACTCCCTT
MZ274341.1_1-1185   CAGATACTAAAGAACTCAGGTAGTGAAGTGCAGGCAGTATTGACCAAGACATACTCCCTT
KC915036.1_54-1238  CAGATACTAAAGAACTCAGGTAGTGAAGTGCAGGCAGTATTGACCAAGACATACTCCCTT
OR365552.1_54-1238  CAGATACTAAAGAACTCAGGTAGTGAGGTGCAGGCAGTATTGACCAAGACATACTCCCTT
OR365551.1_54-1238  CAGATACTAAAGAACTCAGGTAGTGAGGTGCAGGCAGTATTGACCAAGACATACTCCCTT
```

```
                           310       320       330       340       350       360
EF199771.1_54-1238  GGGAAGGGCAAAAAACAGCAAAGGGGAGGAGTTGCAAATGTTAGACATACATGGGGTTGAA
AY028558.1_14-1196  GGGAAGGGCAAAAAACAGCAATGGGGAGGAGTTGCAAATGTTAGACATACATGGGGTTGAA
EF199772.1_54-1238  GGGAAGGGCAAAAAACAGCAAAGGGGAGGAGTTGCAAATGTTAGACATACATGGGGTTGAA
FJ977568.1_54-1238  GGGAAGGGCAAAAAACAGCAAAGGGGAGGAGTTGCAAATGTTAGACATACATGGGGTTGAA
AY028559.1_14-1196  GGGAAGGGCAAAAAACAGCAAAGGGGAGGAGTTGCAAATGTTAGACATACATGGGGTTGAA
AF368173.1_14-1196  GGGAAGGGCAAAAAACAGCAAAGGGGAGGAGTTGCAAATGTTAGACATACATGGGGTTGAA
AY028557.1_14-1195  GGGAAGGGCAAAAAACAGCAAAGGGGAGGAGTTGCAAATGTTAGACATACATGGGGTTGAA
AY590688.1_54-1238  GGGAAGGGCAAAAAACAGCAAAGGGGAGGAGTTGCAAATGTTAGACATACATGGGGTTGAA
AF176590.1_14-1198  GGGAAGGGCAAAAAACAGCAAAGGGGAGGAGTTGCAAATGTTAGACATACATGGGGTTGAA
AY579780.1_54-1238  GGGAAGGGCAAAAAACAGCAAAGGGGAGGAGTTGCAAATGTTAGACATACATGGGGTTGAA
AY028556.1_14-1196  GGGAAGGGCAAAAAACAGCAAAGGGGAGGAGTTGCAAATGTTAGACATACATGGGGTTGAA
AY028568.1_14-1196  GGGAAGGGCAAAAAACAGCAAAGGGGAGGAGTTGCAAATGTTAGACATACATGGGGTTGAA
AY028564.1_14-1196  GGGAAGGGCAAAAAACAGCAAAGGGGAGGAGTTGCAAATGTTAGACATACATGGGGTTGAA
AY028562.1_14-1196  GGGAAGGGCAAAAAACAGCAAAGGGGAGGAGTTGCAAATGTTAGACATACATGGGGTTGAA
AY028561.1_14-1196  GGGAAGGGCAAAAAACAGCAAAGGGGAGGAGTTGCAAATGTTAGACATACATGGGGTTGAA
AY028565.1_14-1196  GGGAAGGGCAAAAAACAGCAAAGGGGAGGAGTTGCAAATGTTAGACATACATGGGGTTGAA
AY028563.1_14-1196  GGGAAGGGCAAAAAACAGCAAAGGGGAGGAGTTGCAAATGTTAGACATACATGGGGTTGAA
AY028567.1_14-1196  GGGAAGGGCAAAAAACAGCAAAGGGGAGGAGTTGCAAATGTTAGACATACATGGGGTTGAA
AY028560.1_14-1196  GGGAAGGGCAAAAAACAGCAAAGGGGAGGAGTTGCAAATGTTAGACATACATGGGGTTGAA
DQ009484.1_14-1198  GGGAAGGGCAAAAAACAGCAAAGGGGAGGAGTTGCAAATGTTAGACATACATGGGGTTGAA
AM293284.1_1-1185   GGTAAAGGCAAAAAACAGTAAAGGGGAGGAATTGCAAATGTTAGACATACATGGGGTTGAA
HG934338.1_55-1239  GGTAAAGGCAAAAAACAGTAAAGGGGAGGAATTGCAAATGTTAGACATACATGGGGTTGAA
PP662686.1_54-1238  GGTAAAGGCAAAAAACAGCAAAGGGGAAGAATTACAAATGTTAGACATACATGGGGTTGAA
OM179883.1_52-1236  GGTAAAGGCAAAAAACAGCAAAGGGGAGGAGTTGCAAATGTTAGACATACATGGGGTGGAA
OM256458.1_54-1238  GGTAAAGGCAAAAAACAGCAAAGGGGAGGAGTTGCAAATGTTAGACATACATGGGGTAGAG
PQ530038.1_1-1185   GGTAAAGGCAAAAAACAGCAAAGGGGAGGATTTGCAAATGTTAGACATACATGGGGTGGAA
MZ274340.1_1-1185   GGTAAAGGCAAAAAACAGCAAAGGGGAGGATTTGCAAATGTTAGACATACATGGGGTGGAA
KF364615.2_14-1198  GGTAAAGGCAAAAAACAGCAAAGGGGAGGATTTGCAAATGTTAGACATACATGGGGTGGAA
MZ274341.1_1-1185   GGTAAAGGCAAAAAACAGCAAAGGGGAGGATTTGCAAATGTTAGACATACATGGGGTGGAA
KC915036.1_54-1238  GGTAAAGGCAAAAAACAGCAAAGGGGAGGATTTGCAAATGTTAGACATACATGGGGTGGAA
OR365552.1_54-1238  GGTAAAGGCAAAAAACAGCAAAGGGGAGGAGTTGCAAATGTTGGACATACATGGGGTGGAG
OR365551.1_54-1238  GGTAAAGGCAAAAAACAGCAAAGGAGAGGAGTTGCAAATGTTGGACATACATGGGGTGGAG
```

```
                            370       380       390       400       410       420
                              .         .         .         .         .         .
EF199771.1_54-1238   AGAAGTTGGATTGAAGAAGTTGACAAAGAGGCAAGGAAAACCATGGCCTCAGCTACAAAG
AY028558.1_14-1196   AGAAGTTGGATTGAAGAAGTTGACAAAGAGGCAAGGAAAACCATGGCCTCAGCTACAAAG
EF199772.1_54-1238   AGAAGTTGGATTGAAGAAGTTGACAAAGAGGCAAGGAAAACCATGGCCTCAGCTACAAAG
FJ977568.1_54-1238   AGAAGTTGGATTGAAGAAGTTGACAAAGAGGCAAGGAAAACCATGGCCTCAGCTACAAAG
AY028559.1_14-1196   AGAAGTTGGATTGAAGAAGTTGACAAAGAGGCAAGGAAAACCATGGCCTCAGCTACAAAG
AF368173.1_14-1196   AGAAGTTGGATTGAAGAAGTTGACAAAGAGGCAAGGAAAACCATGGCCTCAGCTACAAAG
AY028557.1_14-1195   AGAAGTTGGATTGAAGAAGTTGACAAAGAGGCAAGGAAAACCATGGCCTCAGCTACAAAG
AY590688.1_54-1238   AGAAGTTGGATTGAAGAAGTTGACAAAGAGGCAAGGAAAACCATGGCCTCAGCTACAAAG
AF176590.1_14-1198   AGAAGTTGGATTGAAGAAGTTGACAAAGAGGCAAGGAAAACCATGGCCTCAGCTACAAAG
AY579780.1_54-1238   AGAAGTTGGATTGAAGAAGTTGACAAAGAGGCAAGGAAAACCATGGCCTCAGCTACAAAG
AY028556.1_14-1196   AGAAGTTGGATTGAAGAAGTTGACAAAGAGGCAAGGAAAACCATGGCCTCAGCTTCAAAG
AY028568.1_14-1196   AAAAGTTGGGTTGAAGAAGTTGACAAGGAGGCAAGGAAAACCATGGCCTCAGCTACAAAG
AY028564.1_14-1196   AAAAGTTGGGTTGAAGAAGTTGACAAGGAGGCAAGGAAAACCATGGCCTCAGCTACAAAG
AY028562.1_14-1196   AAAAGTTGGGTTGAAGAAGTTGACAAGGAGGCAAGGAAAACCATGGCCTCAGCTACAAAG
AY028561.1_14-1196   AAAAGTTGGGTTGAAGAAGTTGACAAGGAGGCAAGGAAAACCATGGCCTCAGCTACAAAG
AY028565.1_14-1196   AAAAGTTGGGTTGAAGAAGTTGACAAGGAGGCAAGGAAAACCATGGCCTCAGCTACAAAG
AY028563.1_14-1196   AAAAGTTGGGTTGAAGAAGTTGACAAGGAGGCAAGGAAAACCATGGCCTCAGCTACAAAG
AY028567.1_14-1196   AAAAGTTGGGTTGAAGAAGTTGACAAGGAGGCAAGGAAAACCATGGCCTCAGCTACAAAG
AY028560.1_14-1196   AAAAGTTGGGTTGAAGAAGTTGACAAGGAGGCAAGGAAAACCATGGCCTCAGCTACAAAG
DQ009484.1_14-1198   AAAAGTTGGGTTGAAGAAGTTGACAAGGAGGCAAGGAAAACCATGGCCTCAGCTACAAAG
AM293284.1_1-1185    AAAAGTTGGGTTGAAGAAGTTGACAAAGAGGCAAGGAAAACCATGGCCACAGCCACAAAA
HG934338.1_55-1239   AAAAGTTGGGTTGAAGAAGTTGACAAAGAGGCAAGGAAAACCATGGCCACAGCCACAAAA
PP662686.1_54-1238   AAAAGTTGGGTTGAAGAAGTTGACAAAGAGGCAAGGAAAACCATGGCCACAGCCACAAAA
OM179883.1_52-1236   AAAAGTTGGGTTGAAGAAGTTGACAAAGAGGCAAGGAAAACCATGGCCTCAGCCACAAAG
OM256458.1_54-1238   AAAAGTTGGGTTGAAGAAGTTGACAAAGAGGCAAGGAAAACCATGGCTTCAGCCACAAAG
PQ530038.1_1-1185    AAAAGTTGGGTTGAAGAAGTTGACAAAGAAGCAAGGAAAACCATGGCCTCAGCCACAAAG
MZ274340.1_1-1185    AAAAGTTGGGTTGAAGAAGTTGACAAAGAAGCAAGGAATACCATGGCCTCAGCCACAAAG
KF364615.2_14-1198   AAAAGTTGGGTTGAAGAAGTTGACAAAGAAGCAAGGAAAACCATGGCCTCAGCCACAAAG
MZ274341.1_1-1185    AAAAGTTGGGTTGAAGAAGTTGACAAAGAAGCAAGGAAAACCATGGCCTCAGCCACAAAG
KC915036.1_54-1238   AAAAGTTGGGTTGAAGAAGTTGACAAAGAAGCAAGGAAAACCATGGCCTCAGCCACAAAG
OR365552.1_54-1238   AAAAGTTGGGTTGAAGAAGTAGACAAAGAAGCAAGAAAAACCATGGCCTCAGCCACAAAG
OR365551.1_54-1238   AAAAGTTGGGTTGAAGAAGTAGACAAAGAAGCAAGAAAAACCATGGCCTCAGCCACAAAG
```

```
                            430       440       450       460       470       480
                              .         .         .         .         .         .
EF199771.1_54-1238   GACAACTCAGGACCAATACCACAAAATCAAAGACCATCATCCCCGGATGCTCCTATCATA
AY028558.1_14-1196   GACAACTCAGGACCAATACCACAAAATCAAAGACCATCATCCCCGGATGCTCCTATCATA
EF199772.1_54-1238   GACAACTCAGGACCAATACCACAAAATCAAAGACCATCATCCCCGGATGCTCCTATCATA
FJ977568.1_54-1238   GACAACTCAGGACCAATACCACAAAATCAAAGACCATCATCCCCGGATGCTCCTATCATA
AY028559.1_14-1196   GACAACTCAGGACCAATACCACAAAATCAAAGACCATCATCCCCGGATGCTCCTATCATA
AF368173.1_14-1196   GACAACTCAGGACCAATACCACAAAATCAAAGACCATCATCCCCGGATGCTCCTATCATA
AY028557.1_14-1195   GACAACTCAGGACCAATACCACAAAATCAAAGACCATCATCCCCGGATGCTCCTATCATA
AY590688.1_54-1238   GACAACTCAGGACCAATACCACAAAATCAAAGACCATCATCCCCGGATGCTCCTATCATA
AF176590.1_14-1198   GACAACTCAGGACCAATACCACAAAATCAAAGACCATCATCCCCGGATGCTCCTATCATA
AY579780.1_54-1238   GACAACTCAGGACCAATACCACAAAATCAAAGACCATCATCCCCGGATGCTCCTATCATA
AY028556.1_14-1196   GACAACTCAGGACCCATACCACAAAATCAAAGACCATCATCCCCGGATGCTCCTATCATA
AY028568.1_14-1196   GACAACTCAGGACCGATACCACAAAATCAAAGACCATCATCCCCGGATGCTCCTATCATA
AY028564.1_14-1196   GACAACTCAGGACCGATACCACAAAATCAAAGACCATCATCCCCGGATGCTCCTATCATA
AY028562.1_14-1196   GACAACTCAGGACCGATACCACAAGATCAAAGACCATCATCCCCGGATGCTCCTATCATA
AY028561.1_14-1196   GACAACTCAGGACCGATACCACAAAATCAAAGACCATCATCCCCGGATGCTCCTATCATA
AY028565.1_14-1196   GACAACTCAGGACCGATACCACAAAATCAAAGACCATCATCCCCGGATGCTCCTATCATA
AY028563.1_14-1196   GACAACTCAGGACCGATACCACAAAATCAAAGACCATCATCCCCGGATGCTCCTATCATA
AY028567.1_14-1196   GACAACTCAGGACCGATACCACAAAATCAAAGACCATCATCCCCGGATGCTCCTATCATA
AY028560.1_14-1196   GACAACTCAGGACCGATACCACAAAATCAAAGACCATCATCCCCGGATGCTCCTATCATA
DQ009484.1_14-1198   GACAACTCAGGACCGATACCACAAAATCAAAGACCATCATCCCCGGATGCTCCTATCATA
AM293284.1_1-1185    GACAATTCAGGGCCAATACCACAAAATCAAAGACCGTCATCCCCAGATGCTCCTATCATA
HG934338.1_55-1239   GACAATTCAGGGCCAATACCACAAAATCAAAGACCGTCATCCCCAGATGCTCCTATCATA
PP662686.1_54-1238   GACAATTCAGGGCCAATACCACAAAATCAAAGACCGTCATCCCCAGATGCTCCTATCATA
OM179883.1_52-1236   GACAACTCAGGGCCAATACCACAAAATCAAAGACCATCATCCCCAGATGCTCCTATCATA
OM256458.1_54-1238   GACAACTCAGGGCCAATACCACAAAATCAAAGACCATCATCCCCAGATGCTCCTATCATA
PQ530038.1_1-1185    GACAACTCAGGGCCAATACCACAAAATCAAAGACCATCATCCCCAGATGCTCCTATCATA
MZ274340.1_1-1185    GACAACTCAGGGCCAATACCACAAAATCAAAGACCATCATCCCCAGATGCTCCTATCATA
KF364615.2_14-1198   GACAACTCAGGGCCAATACCACAAAATCAAAGACCATCATCCCCAGATGCTCCTATCATA
MZ274341.1_1-1185    GACAACTCAGGGCCAATACCACAAAATCAAAGACCATCATCCCCAGATGCTCCTATCATA
KC915036.1_54-1238   GACAACTCAGGGCCAATACCACAAAATCAAAGACCATCATCCCCAGATGCTCCTATCATA
OR365552.1_54-1238   GACAACTCAGGGCCAATACCACAAAATCAAAGACCATCATCCCCAGATGCTCCTATCATA
OR365551.1_54-1238   GACAACTCAGGGCCAATACCACAAAATCAAAGACCATCATCCCCAGATGCTCCTATCATA
```

```
                              490       500       510       520       530       540
EF199771.1_54-1238    CTACTCTGCATAGGAGCATTAATCTTCACGAAGCTGGCATCAACAATCGAAGTTGGGCTG
AY028558.1_14-1196    CTACTCTGCATAGGAGCATTAATCTTCACGAAGCTGGCATCAACAATCGAAGTTGGGCTG
EF199772.1_54-1238    CTACTCTGCATAGGAGCATTAATCTTCACGAAGCTGGCATCAACAATCGAAGTTGGGCTG
FJ977568.1_54-1238    CTACTCTGCATAGGAGCATTAATCTTCACGAAGCTGGCATCAACAATCGAAGTTGGGCTG
AY028559.1_14-1196    CTACTCTGCATAGGAGCATTAATCTTCACGAAGCTGGCATCAACAATCGAAGTTGGGCTG
AF368173.1_14-1196    CTACTCTGCATAGGAGCATTAATCTTCACGAAGCTGGCATCAACAATCGAAGTTGGGCTG
AY028557.1_14-1195    CTACTCTGCATAGGAGCATTAATCTTCACGAAGCTGGCATCAACAATCGAAGTTGGGCTG
AY590688.1_54-1238    CTACTCTGCATAGGAGCATTAATCTTCACGAAGCTGGCATCAACAATCGAAGTTGGGCTG
AF176590.1_14-1198    CTACTCTGCATAGGAGCATTAATCTTCACGAAGCTGGCATCAACAATCGAAGTTGGGCTG
AY579780.1_54-1238    CTACTCTGCATAGGAGCATTAATCTTCACGAAGCTGGCATCAACAATCGAAGTTGGGCTG
AY028556.1_14-1196    CTACTCTGCATAGGAGCATTAATCTTCACGAAGCTGGCATCAACAATCGAAGTTGGGCTG
AY028568.1_14-1196    CTACTCTGCATAGGAGCATTAATATTCACGAAGCTGGCATCAACAATCGAAGTTGGGCTG
AY028564.1_14-1196    CTACTCTGCATAGGAGCATTAATATTCACGAAGCTGGCATCAACAATCGAAGTTGGGCTG
AY028562.1_14-1196    CTACTCTGCATAGGAGCATTAATATTCACGAAGCTGGCATCAACAATCGAAGTTGGGCTG
AY028561.1_14-1196    CTACTCTGCATAGGAGCATTAATATTCACGAAGCTGGCATCAACAATCGAAGTTGGGCTG
AY028565.1_14-1196    CTACTCTGCATAGGAGCATTAATATTCACGAAGCTGGCATCAACAATCGAAGTTGGGCTG
AY028563.1_14-1196    CTACTCTGCATAGGAGCATTAATATTCACGAAGCTGGCATCAACAATCGAAGTTGGGCTG
AY028567.1_14-1196    CTACTCTGCATAGGAGCATTAATATTCACGAAGCTGGCATCAACAATCGAAGTTGGGCTG
AY028560.1_14-1196    CTACTCTGCATAGGAGCATTAATATTCACGAAGCTGGCATCAACAATCGAAGTTGGGCTG
DQ009484.1_14-1198    CTACTCTGCATAGGAGCATTAATCTTCACGAAGCCGGCATCAACAATCGAAGTTGGGCTG
AM293284.1_1-1185     TTACTCTGCATAGGGGCATTAATCTTCACAAAGCTGGCATCAACAATCGAAGTTGGGCTG
HG934338.1_55-1239    TTACTCTGCATAGGGGCATTAATCTTCACAAAGCTGGCATCAACAATCGAAGTTGGGCTG
PP662686.1_54-1238    TTACTCTGCATAGGAGCATTAATCTTTACAAAGCTGGCATCAACAGTCGAAGTTGGGCTG
OM179883.1_52-1236    TTACTCTGCATAGGAGCATTAATCTTCACAAAGCTGGCATCAACAATCGAAGTTGGACTG
OM256458.1_54-1238    TTACTCTGCATAGGAGCATTAATCTTCACGAAGCTGGCATCAACAATCGAAGTTGGACTG
PQ530038.1_1-1185     TTACTCTGCATAGGAGCATTAATCTTCACGAAGCTGGCATCAACAATCGAAGTTGGACTG
MZ274340.1_1-1185     TTACTCTGCATAGGAGCATTAATCTTCACGAAGCTGGCATCAACAATCGAAGTTGGACTG
KF364615.2_14-1198    TTACTCTGCATAGGAGCATTAATCTTCACGAAGCTGGCATCAACAATCGAAGTTGGACTG
MZ274341.1_1-1185     TTACTCTGCATAGGAGCATTAATCTTCACGAAGCTGGCATCAACAATCGAAGTTGGACTG
KC915036.1_54-1238    TTACTCTGCATAGGAGCATTAATCTTCACGAAGCTGGCATCAACAATCGAAGTTGGACTG
OR365552.1_54-1238    TTACTCTGCATAGGAGCATTAATCTTCACGAAGCTGGCATCAACAATCGAAGTTGGACTG
OR365551.1_54-1238    TTACTCTGCATAGGAGCATTAATCTTCACGAAGCTGGCATCAACAATCGAAGTTGGACTG
```

```
                              550       560       570       580       590       600
EF199771.1_54-1238    GAGACAGCTGTTAGAAGGGCGAACCGTGTGCTGAGTGATGCATTGAAAAGGTTCCCAAGG
AY028558.1_14-1196    GAGACAGCTGTTAGAAGGGCGAACCGTGTGCTGAGTGATGCATTGAAAAGGTTCCCAAGG
EF199772.1_54-1238    GAGACAGCTGTTAGAAGGGCGAACCGTGTGCTGAGTGATGCATTGAAAAGGTTCCCAAGG
FJ977568.1_54-1238    GAGACAGCTGTTAGAAGGGCGAACCGTGTGCTGAGTGATGCATTGAAAAGGTTCCCAAGG
AY028559.1_14-1196    GAGACAGCTGTTAGAAGGGCGAACCGTGTGCTGAGTGATGCATTGAAAAGGTTCCCAAGG
AF368173.1_14-1196    GAGACAGCTGTTAGAAGGGCAAACCGTGTGCTGAATGATGCATTGAAAAGGTTCCCAAGG
AY028557.1_14-1195    GAGACAGCTGTTAGAAGGGCAAACCGTGTGCTGAATGATGCACTGAGAAGGTTCCCAAGG
AY590688.1_54-1238    GAGACAGCTGTTAGAAGGGCAAACCGTGTGCTGAATGATGCATTGAAAAGGTTCCCAAGG
AF176590.1_14-1198    GAGACAGCTGTTAGAAGGGCAAACCGTGTGCTGAATGATGCATTGAAAAGGTTCCCAAGG
AY579780.1_54-1238    GAGACAGCTGTTAGAAGGGCAAACCGTGTGCTGAATGATGCATTGAAAAGGTTCCCAAGG
AY028556.1_14-1196    GAGACAGCTGTTAGAAGGGCAAACCGTGTGCTGAATGATGCATTGAAAAGGTTCCCAAGG
AY028568.1_14-1196    GAGACAGCTGTTAGAAGGGCAAACCGCGTGCTGAATGATGCATTGAAAAGGTTCCCAAGG
AY028564.1_14-1196    GAGACAGCTGTTAGAAGGGCAAACCGCGTGCTGAATGATGCATTGAAAAGGTTCCCAAGG
AY028562.1_14-1196    GAGACAGCTGTTAGAAGGGCAAACCGCGTGCTGAATGATGCATTGAAAAGGTTCCCAAGG
AY028561.1_14-1196    GAGACAGCTGTTAGAAGGGCAAACCGCGTGCTGAATGATGCATTGAAAAGGTTCCCAAGG
AY028565.1_14-1196    GAGACAGCTGTTAGAAGGGCAAACCGCGTGCTGAATGATGCATTGAAAAGGTTCCCAAGG
AY028563.1_14-1196    GAGACAGCTGTTAGAAGGGCAAACCGCGTGCTGAATGATGCATTGAAAAGGTTCCCAAGG
AY028567.1_14-1196    GAGACAGCTGTTAGAAGGGCAAACCGCGTGCTGAATGATGCATTGAAAAGGTTCCCAAGG
AY028560.1_14-1196    GAGACAGCTGTTAGAAGGGCAACCCGCGTGCTGAATGATGCATTGAAAAGGTTCCCAAGG
DQ009484.1_14-1198    GAGACAGCTGTTAGAAGGGCAAACCGCGTGCTGAATGATGCATTGAAAAGGTTCCCAAGG
AM293284.1_1-1185     GAGACAGCTGTTAGAAGGGCAAACCGTGTGCTGAATGATGCATTGAAAAGGTTCCCTAGG
HG934338.1_55-1239    GAGACAGCTGTTAGAAGGGCAAACCGTGTGCTGAATGATGCATTGAAAAGGTTCCCTAGG
PP662686.1_54-1238    GAGACAGCTGTTAGAAGGGCAAACCGTGTGCTGAATGATGCATTGAAAAGGTTTCCAAGG
OM179883.1_52-1236    GAGACGGCTGTCAGAAGGGCAAACCGTGTGCTGAATGATGCATTGAAAAGGTTCCCGAGG
OM256458.1_54-1238    GAGACAGCTGTCAGAAGGGCAAACCGTGTGCTGAATGATGCATTGAAAAGGTTCCCGAGG
PQ530038.1_1-1185     GAGACAGCTGTTAGAAGGGCAAACCGTGTGCTGAATGATGCATTAAAGAGGTTCCCGAGG
MZ274340.1_1-1185     GAGACAGCTGTTAGAAGGGCAAACCGTGTGCTGAATGATGCATTAAAGAGGTTCCCGAGG
KF364615.2_14-1198    GAGACAGCTGTTAGAAGGGCAAACCGTGTGCTGAATGATGCATTAAAGAGGTTCCCGAGG
MZ274341.1_1-1185     GAGACAGCTGTTAGAAGGGCAAACCGTGTGCTGAATGATGCATTAAAGAGGTTCCCGAGG
KC915036.1_54-1238    GAGACAGCTGTTAGAAGGGCAAACCGTGTGCTGAATGATGCATTAAAGAGGTTCCCGAGG
OR365552.1_54-1238    GAGACAGCTGTTAGAAGGGCAAACCGTGTGCTGAATGATGCATTAAAAAGGTTCCCGAGG
OR365551.1_54-1238    GAGACAGCTGTTAGAAGGGCAAACCGTGTGCTGAATGATGCATTAAAAAGGTTCCCGAGG
```

```
                            610       620       630       640       650       660
EF199771.1_54-1238   ATTGACATCCCCAAAATTGCGAGGTCCTTTTATGATCTGTTTGAGCAGAAAGTTTACTAC
AY028558.1_14-1196   ATTGACATCCCCAAAATTGCGAGGTCCTTTTATGATCTGTTTGAGCAGAAAGTTTACTAC
EF199772.1_54-1238   ATTGACATCCCCAAAATTGCGAGGTCCTTTTATGATCTGTTTGAGCAGAAAGTTTACTAC
FJ977568.1_54-1238   ATTGACATCCCCAAAATTGCGAGGTCCTTTTATGATCTGTTTGAGCAGAAAGTTTACTAC
AY028559.1_14-1196   ATTGACATCCCCAAAATTGCGAGGTCCTTTTATGATCTGTTTGAGCAGAAAGTTTACTAC
AF368173.1_14-1196   ATTGACATCCCCAAAATTGCGAGGTCCTTTTATGATCTGTTTGAGCAGAAAGTTTACTAC
AY028557.1_14-1195   ATTGACATCCCCAAAATTGCGAGGTCCTTTTATGATCTGTTTGAGCAGAAAGTTTACTAC
AY590688.1_54-1238   ATTGACATCCCCAAAATTGCGAGGTCCTTTTATGATCTGTTTGAGCAGAAAGTTTACTAC
AF176590.1_14-1198   ATTGACATCCCCAAAATTGCGAGGTCCTTTTATGATCTGTTTGAGCAGAAAGTTTACTAC
AY579780.1_54-1238   ATTGACATCCCCAAAATTGCGAGGTCCTTTTATGATCTGTTTGAGCAGAAAGTTTACTAC
AY028556.1_14-1196   ATTGACATCCCCAAAATTGCAAGGTCCTTTTATGATCTGTTTGAGCAGAAAGTTTACTAC
AY028568.1_14-1196   ATCGACATCCCCAAAATTGCAAGGTCCTTTTATGATCTGTTTGAGCAGAAAGTTTACTAT
AY028564.1_14-1196   ATCGACATCCCCAAAATTGCAAGGTCCTTTTATGATCTGTTTGAGCAGAAAGTTTACTAT
AY028562.1_14-1196   ATCGACATCCCCAAAATTGCAAGGTCCTTTTATGATCTGTTTGAGCAGAAAGTTTACTAT
AY028561.1_14-1196   ATCGACATCCCCAAAATTGCAAGGTCCTTTTATGATCTGTTTGAGCAGAAAGTTTACTAT
AY028565.1_14-1196   ATCGACATCCCCAAAATTGCAAGGTCCTTTTATGATCTGTTTGAGCAGAAAGTTTACTAT
AY028563.1_14-1196   ATCGACATCCCCAAAATTGCAAGGTCCTTTTATGATCTGTTTGAGCAGAAAGTTTACTAT
AY028567.1_14-1196   ATCGACATCCCCAAAATTGCAAGGTCCTTTTATGATCTGTTTGAGCAGAAAGTTTACTAT
AY028560.1_14-1196   ATCGACATCCCCAAAATTGCAAGGTCCTTTTATGATCTGTTTGAGCAGAAAGTTTACTAT
DQ009484.1_14-1198   ATCGACATCCCCAAAATTGCGAGGTCCTTTTATGATCTGTTTGAGCAGAAAGTTTACTAT
AM293284.1_1-1185    ATTGACATTCCCAAAATTGCAAGGTCCTTCTATGATCTGTTTGAACAGAAAGTTTACTAC
HG934338.1_55-1239   ATTGACATTCCCAAAATTGCAAGGTCCTTCTATGATCTGTTTGAACAGAAAGTTTACTAC
PP662686.1_54-1238   ATTGACATCCCCAAAATTGCGAGGTCCTTTTATGATCTGTTTGAACAGAAAGTTTATTAC
OM179883.1_52-1236   ATTGACATTCCCAAAATTGCGAGGTCCTTTTATGATCTGTTTGAACAGAAAGTTTATTAC
OM256458.1_54-1238   ATTGACATCCCCAAAATTGCGAGGTCCTTTTATGATCTGTTTGAACAGAAAGTTTATTAC
PQ530038.1_1-1185    ATTGACATCCCCAAAATTGCGAGGTCCTTTTATGATCTGTTTGAACAGAAAGTTTATTAC
MZ274340.1_1-1185    ATTGACATCCCCAAAATTGCGAGGTCCTTTTATGATCTGTTTGAACAGAAAGTTTATTAC
KF364615.2_14-1198   ATTGACATCCCCAAAATTGCGAGGTCCTTTTATGATCTGTTTGAACAGAAAGTTTATTAC
MZ274341.1_1-1185    ATTGACATCCCCAAAATTGCGAGGTCCTTTTATGATCTGTTTGAACAGAAAGTTTATTAC
KC915036.1_54-1238   ATTGACATCCCCAAAATTGCGAGGTCCTTTTATGATCTGTTTGAACAGAAAGTTTATTAC
OR365552.1_54-1238   ATTGACATCCCCAAAATTGCGAGGTCCTTTTATGATCTGTTTGAACAGAAAGTTTATTAC
OR365551.1_54-1238   ATTGACATCCCCAAAATTGCGAGGTCCTTTTATGATCTGTTTGAACAGAAAGTTTATTAC
```

```
                            670       680       690       700       710       720
EF199771.1_54-1238   AGGAGCTTGTTTATAGAGTATGGCAAAGCCCTTGGGTCTTCTTCCACAGGAAGCAAGGCA
AY028558.1_14-1196   AGGAGCTTGTTCATAGAGTATGGCAAAGCCCTTGGGTCTTCTTCCACAGGAAGCAAGGCA
EF199772.1_54-1238   AGGAGCTTGTTTATAGAGTATGGCAAAGCCCTTGGGTCTTCTTCCACAGGAAGCAAGGCA
FJ977568.1_54-1238   AGGAGCTTGTTTATAGAGTATGGCAAAGCCCTTGGGTCTTCTTCCACAGGAAGCAAGGCA
AY028559.1_14-1196   AGGAGCTTGTTTATAGAGTATGGCAAAGCCCTTGGGTCTTCTTCCACAGGAAGCAAGGCA
AF368173.1_14-1196   AGGAGCTTGTTTATAGAGTATGGCAAAGCCCTTGGGTCTTCTTCCACAGGAAGCAAGGCA
AY028557.1_14-1195   AGGAGCTTGTTTATAGAGTATGGCAAAGCCCTTGGGTCTTCTTCCACAGGAAGCAAGGCA
AY590688.1_54-1238   AGGAGCTTGTTTATAGAGTATGGCAAAGCCCTTGGGTCTTCTTCCACAGGAAGCAAGGCA
AF176590.1_14-1198   AGGAGCTTGTTTATAGAGTATGGCAAAGCCCTTGGGTCTTCTTCCACAGGAAGCAAGGCA
AY579780.1_54-1238   AGGAGCTTGTTTATAGAGTATGGCAAAGCCCTTGGGTCTTCTTCCACAGGAAGCAAGGCA
AY028556.1_14-1196   AGGAGCTTGTTTATAGAGTATGGCAAAGCCCTTGGGTCTTCTTCCACAGGAAGCAAGGCA
AY028568.1_14-1196   AGGAGCTTGTTTATAGAGTATGGCAAAGCCCTTGGGTCTTCTTCCACAGGAAGCAAGGCA
AY028564.1_14-1196   AGGAGCTTGGTTATAGAGTATGGCAAAGCCCTTGGGTCTTCTTCCACAGGAAGCAAGGCA
AY028562.1_14-1196   AGGAGCTTGTTTATAGAGTATGGCAAAGCCCTTGGGTCTTCTTCCACAGGAAGCAAGGCA
AY028561.1_14-1196   AGGAGCTTGTTTATAGAGTATGGCAAAACCCTTGGGTCTTCTTCCACAGGAAGCAAGGCA
AY028565.1_14-1196   AGGAGCTTGTTTATAGAGTATGGCAAAGCCCTTGGGTCTTCTTCCACAGGAAGCAAGGCA
AY028563.1_14-1196   AGGAGCTTGTTTATAGAGTATGGCAAAGCCCTTGGGTCTTCTTCCACAGGAAGCAAGGCA
AY028567.1_14-1196   AGGAGCTTGTTTATAGAGTATGGCAAAGCCCTTGGGTCTTCTTCCACAGGAAGCAAGGCA
AY028560.1_14-1196   AGGAGCTTGTTTATAGAGTATGGCAAAGCCCTTGGGTCTTCTTCCACAGGAAGCAAGGCA
DQ009484.1_14-1198   AGGAGCTTGTTTATAGAGTATGGCAAAGCCCTTGGGTCTTCTTCCACAGGAAGCAAGGCA
AM293284.1_1-1185    AGGAGCTTGTTTATAGAGTATGGCAAGGCCCTTGGGTCTTCTTCCACAGGAAGCAAGGCA
HG934338.1_55-1239   AGGAGCTTGTTTATAGAGTATGGCAAGGCCCTTGGGTCTTCTTCCACAGGAAGCAAGGCA
PP662686.1_54-1238   AGGAGCTTGTTTATAGAGTATGGCAAAGCCCTAGGATCTTCTTCCACAGGAAGCAAGGCA
OM179883.1_52-1236   AGGAGCTTGTTTATAGAGTATGGCAAAGCCCTTGGGTCTTCTTCCACGGGAAGCAAGGCA
OM256458.1_54-1238   AGGAGCTTGTTTATAGAGTATGGCAAAGCCCTTGGGTCTTCTTCCACGGGAAGCAAGGCA
PQ530038.1_1-1185    AGGAGCTTGTTTATAGAGTATGGCAAAGCCCTTGGGTCTTCTTCCACGGGAAGCAAAGCA
MZ274340.1_1-1185    AGGAGCTTGTTTATAGAGTATGGCAAAGCCCTTGGGTCTTCTTCCACGGGAAGCAAAGCA
KF364615.2_14-1198   AGGAGCTTGTTTATAGAGTATGGCAAAGCCCTTGGGTCTTCTTCCACGGGAAGCAAAGCA
MZ274341.1_1-1185    AGGAGCTTGTTTATAGAGTACGGCAAAGCCCTTGGGTCTTCTTCCACGGGAAGCAAAGCA
KC915036.1_54-1238   AGGAGCTTGTTTATAGAGTATGGCAAAGCCCTTGGGTCTTCTTCCACGGGAAGCAAAGCA
OR365552.1_54-1238   AGGAGCTTGTTTATAGAGTATGGCAAAGCCCTTGGGTCTTCTTCCACGGGAAGCAAGGCA
OR365551.1_54-1238   AGGAGCTTGTTTATAGAGTATGGCAAAGCCCTTGGGTCTTCTTCCACGGGAAGCAAGGCA
```

```
                                 730       740       750       760       770       780
                                   .         .         .         .         .         .
EF199771.1_54-1238    GAAAGCCTGTTTGTGAATATTTTCATGCAAGCTTATGGTGCAGGTCAGACAATGCTAAGA
AY028558.1_14-1196    GAAAGCCTGTTTGTGAATATTTTCATGCAAGCTTATGGTGCAGGTCAGACAATGCTAAGA
EF199772.1_54-1238    GAAAGCCTGTTTGTGAATATTTTCATGCAAGCTTATGGTGCAGGTCAGACAATGCTAAGA
FJ977568.1_54-1238    GAAAGCCTGTTTGTGAATATTTTCATGCAAGCTTATGGTGCAGGTCAGACAATGCTAAGA
AY028559.1_14-1196    GAAAGCCTGTTTGTGAATATTTTCATGCAAGCTTATGGTGCAGGTCAGACAATGCTAAGA
AF368173.1_14-1196    GAAAGCCTGTTTGTGAATATTTTCATGCAAGCTTATGGTGCAGGTCAGACAATGCTAAGA
AY028557.1_14-1195    GAAAGCCTGTTTGTGAATATTTTCATGCAAGCTTATGGTGCAGGTCAGACAATGCTAAGA
AY590688.1_54-1238    GAAAGCCTGTTTGTGAATATTTTCATGCAAGCTTATGGTGCAGGTCAGACAATGCTAAGA
AF176590.1_14-1198    GAAAGCCTGTTTGTGAATATTTTCATGCAAGCTTATGGTGCAGGTCAGACAATGCTAAGA
AY579780.1_54-1238    GAAAGCCTGTTTGTGAATATTTTCATGCAAGCTTATGGTGCAGGTCAGACAATGCTAAGA
AY028556.1_14-1196    GAAAGCCTGTTTGTGAATATTTTCATGCAAGCTTATGGTGCAGGTCAGACAATGCTAAGA
AY028568.1_14-1196    GAAAGTCTGTTTGTGAACATTTTCATGCAAGCTTATGGTGCAGGTCAGACAATGCTAAGG
AY028564.1_14-1196    GAAAGTCTGTTTGTGAACATTTTCATGCAAGCTTATGGTGCAGGTCAGACAATGCTAAGG
AY028562.1_14-1196    GAAAGTCTGTTTGTGAACATTTTCATGCAAGCTTATGGTGCAGGTCAGACAATGCTAAGG
AY028561.1_14-1196    GAAAGTCTGTTTGTGAACATTTTCATGCAAGCTTATGGTGCAGGTCAGACAATGCTAAGG
AY028565.1_14-1196    GAAAGTCTGTTTGTGAACATTTTCATGCAAGCTTATGGTGCAGGTCAGACAATGCTAAGG
AY028563.1_14-1196    GAAAGTCTGTTTGTGAACATTTTCATGCAAGCTTATGGTGCAGGTCAGACAATGCTAAGG
AY028567.1_14-1196    GAAAGTCTGTTTGTGAACATTTTCATGCAAGCTTATGGTGCAGGTCAGACAATGCTAAGG
AY028560.1_14-1196    GAAAGTCTGTTTGTGAACATTTTCATGCAAGCTTATGGTGCAGGTCAGACAATGCTAAGG
DQ009484.1_14-1198    GAAAGCCTGTTTGTGAACATTTTCATGCAAGCTTATGGTGCAGGTCAGACAATGCTAAGG
AM293284.1_1-1185     GAGAGTCTGTTTGTGAACATTTTCATGCAAGCTTATGGTGCAGGCCAAACAATGCTAAGG
HG934338.1_55-1239    GAGAGTCTGTTTGTGAACATTTTCATGCAAGCTTATGGTGCAGGCCAAACAATGCTAAGG
PP662686.1_54-1238    GAGAGCCTGTTTGTAAACATTTTCATGCAGGCTTATGGTGCAGGCCAAACAATGCTAAGG
OM179883.1_52-1236    GAGAGCCTGTTTGTGAACATTTTCATGCAAGCTTATGGTGCAGGACAAACAATGCTAAGG
OM256458.1_54-1238    GAGAGCCTGTTTGTGAACATTTTCATGCAAGCTTATGGTGCAGGACAAACAATGCTAAGG
PQ530038.1_1-1185     GAGAGCCTGTTTGTGAACATTTTCATGCAAGCTTATGGTGCAGGCCAAACAATGCTAAGG
MZ274340.1_1-1185     GAGAGCCTGTTTGTGAACATTTTCATGCAAGCTTATGGTGCAGGCCAAACAATGCTAAGG
KF364615.2_14-1198    GAGAGCCTGTTTGTGAACATTTTCATGCAAGCTTATGGTGCAGGCCAAACAATGCTAAGG
MZ274341.1_1-1185     GAGAGCCTGTTTGTGAACATTTTCATGCAAGCTTATGGTGCAGGCCAAACAATGCTAAGG
KC915036.1_54-1238    GAGAGCCTGTTTGTGAACATTTTCATGCAAGCTTATGGTGCAGGCCAAACAATGCTAAGG
OR365552.1_54-1238    GAGAGCCTGTTTGTGAACATTTTCATGCAAGCTTATGGTGCAGGCCAAACAATGCTAAGG
OR365551.1_54-1238    GAGAGCCTGTTTGTGAACATTTTCATGCAAGCTTATGGTGCAGGCCAAACAATGCTAAGG
```

```
                                 790       800       810       820       830       840
                                   .         .         .         .         .         .
EF199771.1_54-1238    TGGGGGGTAATTGCCAGATCATCCAACAATATAATGTTGGGTCATGTCTCCGTACAAGCA
AY028558.1_14-1196    TGGGGGGTAATTGCCAGATCATCCAACAATATAATGTTGGGTCATGTCTCCGTACAAGCA
EF199772.1_54-1238    TGGGGGGTAATTGCCAGATCATCCAACAATATAATGTTGGGTCATGTCTCCGTACAAGCA
FJ977568.1_54-1238    TGGGGGGTAATTGCCAGATCATCCAACAATATAATGTTGGGTCATGTCTCCGTACAAGCA
AY028559.1_14-1196    TGGGGGGTAATTGCCAGATCATCCAACAATATAATGTTGGGTCATGTCTCCGTACAAGCA
AF368173.1_14-1196    TGGGGGGTAATTGCCAGATCATCCAACAATATAATGTTGGGCCATGTCTCCGTACAAGCA
AY028557.1_14-1195    TGGGGGGTAATTGCCAGATCATCCAACAATATAATGTTGGGCCATGTCTCCGTACAAGCA
AY590688.1_54-1238    TGGGGGGTAATTGCCAGATCATCCAACAATATAATGTTGGGCCATGTCTCCGTACAAGCA
AF176590.1_14-1198    TGGGGGGTAATTGCCAGATCATCCAACAATATAATGTTGGGCCATGTCTCCGTACAAGCA
AY579780.1_54-1238    TGGGGGGTAATTGCCAGATCATCCAACAATATAATGTTGGGCCATGTCTCCGTACAAGCA
AY028556.1_14-1196    TGGGGGGTAATTGCCAGATCATCCAACAATATAATGTTGGGCCATGTCTCCGTACAAGCA
AY028568.1_14-1196    TGGGGGGTAATTGCCAGATCATCCAACAATATAATGTTGGGCCATGTCTCCGTACAAGCG
AY028564.1_14-1196    TGGGGGGTAATTGCCAGATCATCCAACAATATAATGTTGGGCCATGTCTCCGTACAAGCG
AY028562.1_14-1196    TGGGGGGTAATTGCCAGATCATCCAACAATATAATGTTGGGCCATGTCTCCGTACAAGCG
AY028561.1_14-1196    TGGGGGGTAATTGCCAGATCATCCAACAATATAATGTTGGGCCATGTCTCCGTACAAGCG
AY028565.1_14-1196    TGGGGGGTAATTGCCAGATCATCCAACAATATAATGTTGGGCCATGTCTCCGTACAAGCG
AY028563.1_14-1196    TGGGGGGTAATTGCCAGATCATCCAACAATATAATGTTGGGCCATGTCTCCGTACAAGCG
AY028567.1_14-1196    TGGGGGGTAATTGCCAGATCATCCAACAATATAATGTTGGGCCATGTCTCCGTACAAGCG
AY028560.1_14-1196    TGGGGGGTAATTGCCAGATCATCCAACAATATAATGTTGGGCCATGTCTCCGTACAAGCG
DQ009484.1_14-1198    TGGGGGGCAATTGCCAGATCATCCAACAATATAATGTTGGGCCATGTCTCCGTACAAGCG
AM293284.1_1-1185     TGGGGAGTGATCGCCAGATCTTCCAACAATATAATGTTAGGCCATGTCTCTGTACAAGCG
HG934338.1_55-1239    TGGGGAGTGATCGCCAGATCTTCCAACAATATAATGTTAGGCCATGTCTCTGTACAAGCG
PP662686.1_54-1238    TGGGGAGTGATCGCTAGATCTTCCAATAATATAATGTTAGGCCATGTCTCTGTACAAGCG
OM179883.1_52-1236    TGGGGAGTGATCGCAAGATCTTCCAACAATATAATGTTGGGCCATGTCTCTGTACAAGCA
OM256458.1_54-1238    TGGGGAGTGATCGCAAGATCTTCCAACAATATAATGTTGGGCCATGTCTCTGTACAAGCA
PQ530038.1_1-1185     TGGGGAGTGATCGCAAGATCTTCCAACAATATAATGTTGGGCCATGTCTCTGTACAAGCA
MZ274340.1_1-1185     TGGGGAGTGATCGCAAGATCTTCCAACAATATAATGTTGGGCCATGTCTCTGTACAAGCA
KF364615.2_14-1198    TGGGGAGTGATCGCAAGATCTTCCAACAATATAATGTTGGGCCATGTCTCTGTACAAGCA
MZ274341.1_1-1185     TGGGGAGTGATCGCAAGATCTTCCAACAATATAATGTTGGGCCATGTCTCTGTACAAGCA
KC915036.1_54-1238    TGGGGAGTGATCGCCAGATCATCCAACAATATAATGTTGGGCCATGTCTCCGTACAAGCA
OR365552.1_54-1238    TGGGGAGTGATCGCAAGATCTTCCAACAATATAATGTTGGGACATGTCTCTGTACAAGCA
OR365551.1_54-1238    TGGGGAGTGATCGCAAGATCTTCCAACAATATAATGTTGGGACATGTCTCTGTACAAGCA
```

```
                                850       860       870       880       890       900
EF199771.1_54-1238  GAACTCAAACAGGTTACGGAGGTATATGATCTAGTTAGAGAGATGGCCCTGAGTCAGGT
AY028558.1_14-1196  GAACTCAAACAGGTTACGGAGGTATATGATCTAGTTAGAGAGATGGCCCTGAGTCAGGT
EF199772.1_54-1238  GAACTCAAACAGGTTACGGAGGTATATGATCTAGTTAGAGAGATGGCCCTGAGTCAGGT
FJ977568.1_54-1238  GAACTCAAACAGGTTACGGAGGTATATGATCTAGTTAGAGAGATGGCCCTGAGTCAGGT
AY028559.1_14-1196  GAACTCAAACAGGTTACGGAGGTATATGATCTAGTTAGAGAGATGGCCCTGAGTCAGGT
AF368173.1_14-1196  GAACTCAAACAGGTTACGGAGGTATATGATCTAGTTAGAGAGATGGCCCTGAGTCAGGT
AY028557.1_14-1195  GAACTCAAACAGGTTACGGAGGTATATGATCTAGTTAGAGAGATGGCCCTGAGTCAGGT
AY590688.1_54-1238  GAACTCAAACAGGTTACGGAGGTATATGATCTAGTTAGAGAGATGGCCCTGAGTCAGGT
AF176590.1_14-1198  GAACTCAAACAGGTTACGGAGGTATATGATCTAGTTAGAGAGATGGCCCTGAGTCAGGT
AY579780.1_54-1238  GAACTCAAACAGGTTACGGAGGTATATGATCTAGTTAGAGAGATGGCCCTGAGTCAGGT
AY028556.1_14-1196  GAACTCAAACAGGTTACGGAGGTATATGATCTAGTTAGAGAGATGGCCCTGAGTCAGGT
AY028568.1_14-1196  GAACTCAAACAGGTCACGGAGGTATATGATCTAGTCAGAGAGATGGCCCTGAGTCAGGT
AY028564.1_14-1196  GAACTCAAACAGGTCACGGAGGTATATGATCTAGTCAGAGAGATGGCCCTGAGTCAGGT
AY028562.1_14-1196  GAACTCAAACAGGTCACGGAGGTATATGATCTAGTCAGAGAGATGGCCCTGAGTCAGGT
AY028561.1_14-1196  GAACTCAAACAGGTCACGGAGGTATATGATCTAGTCAGAGAGATGGCCCTGAGTCAGGT
AY028565.1_14-1196  GAACTCAAACAGGTCACGGAGGTATATGATCTAGTCAGAGAGATGGCCCTGAGTCAGGT
AY028563.1_14-1196  GAACTCAAACAGGTCACGGAGGTATATGATCTAGTCAGAGAGATGGCCCTGAGTCAGGT
AY028567.1_14-1196  GAACTCAAACAGGTCACGGAGGTATATGATCTAGTCAGAGAGATGGCCCTGAGTCAGGT
AY028560.1_14-1196  GAACTCAAACAGGTCACGGAGGTATATGATCTAGTCAGAGAGATGGCCCTGAGTCAGGT
DQ009484.1_14-1198  GAACTCAAACAGGTCACGGAGGTATATGATCTAGTCAGAGAGATGGCCCTGAGTCAGGT
AM293284.1_1-1185   GAACTCAAACAGGTCACGGAGGTATATGATCTAGTTAGAGAGATGGCCCCGAGTCAGGT
HG934338.1_55-1239  GAACTCAAACAGGTCACGGAGGTATATGATCTAGTTAGAGAGATGGCCCCGAGTCAGGT
PP662686.1_54-1238  GAACTCAAACAGGTCACGGAGGTATATGACCTAGTTAGAGAAATGGCCCTGAGTCAGGT
OM179883.1_52-1236  GAACTCAAACAGGTCACGGAGGTATATGATCTAGTTAGAGAAATGGCCCTGAGTCAGGT
OM256458.1_54-1238  GAACTCAAACAGGTCACGGAGGTATATGATCTAGTTAGAGAAATGGCCCTGAGTCAGGT
PQ530038.1_1-1185   GAACTCAAACAGGTCACGGAGGTATATGATCTGGTTAGAGAAATGGCCCTGAGTCAGGT
MZ274340.1_1-1185   GAACTCAAACAGGTCACGGAGGTATATGATCTAGTTAGAGAAATGGCCCTGAGTCAGGT
KF364615.2_14-1198  GAACTCAAACAGGTCACGGAGGTATATGATCTAGTTAGAGAAATGGCCCTGAGTCAGGT
MZ274341.1_1-1185   GAACTCAAACAGGTCACGGAGGTATATGATCTAGTTAGAGAAATGGCCCTGAGTCAGGT
KC915036.1_54-1238  GAACTCAAACAGGTCACGGAGGTATATGATCTAGTTAGAGAAATGGCCCTGAGTCAGGT
OR365552.1_54-1238  GAACTCAAACAGGTCACGGAGGTATATGATCTAGTTAGAGAAATGGCCCTGAGTCAGGT
OR365551.1_54-1238  GAACTCAAACAGGTCACGGAGGTATATGATCTAGTTAGAGAAATGGCCCTGAGTCAGGT
```

```
                                910       920       930       940       950       960
EF199771.1_54-1238  CTTCTTCACCTGAGGCAAAGCCCTAAGGCTGGGTTGTTGTCACTTGCTAATTGTCCCAAT
AY028558.1_14-1196  CTTCTTCACCTGAGGCAAAGCCCTAAGGCTGGGTTGTTGTCACTTGCTAATTGTCCCAAT
EF199772.1_54-1238  CTTCTTCACCTGAGGCAAAGCCCTAAGGCTGGGTTGTTGTCACTTGCTAATTGTCCCAAT
FJ977568.1_54-1238  CTTCTTCACCTGAGGCAAAGCCCTAAGGCTGGGTTGTTGTCACTTGCTAATTGTCCCAAT
AY028559.1_14-1196  CTTCTTCACCTGAGGCAAAGCCCTAAGGCTGGGTTGTTGTCACTTGCTAATTGTCCCAAT
AF368173.1_14-1196  CTTCTTCACCTGAGGCAAAGCCCTAAGGCAGGGTTGTTGTCACTTGCCAATTGTCCCAAT
AY028557.1_14-1195  CTTCTTCACCTGAGGCAAAGCCCTAAGGCAGGGTTGTTGTCACTTGCCAATTGTCCCAAT
AY590688.1_54-1238  CTTCTTCACCTGAGGCAAAACCCTAAGGCAGGGTTGTTGTCACTTGCCAATTGTCCCAAT
AF176590.1_14-1198  CTTCTTCACCTGAGGCAAAACCCTAAGGCAGGGTTGTTGTCACTTGCCAATTGTCCCAAT
AY579780.1_54-1238  CTTCTTCACCTGAGGCAAAACCCTAAGGCAGGGTTGTTGTCACTTGCCAATTGTCCCAAT
AY028556.1_14-1196  CTTCTTCACCTGAGGCAAAGCCCTAAGGCAGGGTTGTTGTCACTTGCCAATTGTCCCAAT
AY028568.1_14-1196  CTTCTCCACCTGAGGCAAAGCCCTAAGGCTGGATTGTTGTCACTTGCTAATTGTCCAAAT
AY028564.1_14-1196  CTTCTCCACCTGAGGCAAAGCCCTAAGGCTGGATTGTTGTCACTTGCTAATTGTCCAAAT
AY028562.1_14-1196  CTTCTCCACCTGAGGCAAAGCCCTAAGGCTGGATTGTTGTCACTTGCTAATTGTCCAAAT
AY028561.1_14-1196  CTTCTCCACCTGAGGCAAAGCCCTAAGGCTGGATTGTTGTCACTTGCTAATTGTCCAAAT
AY028565.1_14-1196  CTTCTCCACCTGAGGCAAAGCCCTAAGGCTGGATTGTTGTCACTTGCTAATTGTCCAAAT
AY028563.1_14-1196  CTTCTCCACCTGAGGCAAAGCCCTAAGGCTGGATTGTTGTCACTTGCTAATTGTCCAAAT
AY028567.1_14-1196  CTTCTCCACCTGAGGCAAAGCCCTAAGGCTGGATTGTTGTCACTTGCTAATTGTCCAAAT
AY028560.1_14-1196  CTTCTCCACCTGAGGCAAAGCCCTAAGGCTGGGTTGTTGTCACTTGCTAATTGTCCAAAT
DQ009484.1_14-1198  CTTCTCCACCTGAGGCAAAGCCCTAAGGCTGGATTGTTGTCACTTGCTAATTGTCCAAAT
AM293284.1_1-1185   CTTCTCCACCTGAGGCAAAGCCCCAAGGCTGGGTTGTTATCACTTGCCAATTGTCCAAAT
HG934338.1_55-1239  CTTCTCCACCTGAGGCAAAGCCCCAAGGCTGGGTTGTTATCACTTGCCAATTGTCCAAAT
PP662686.1_54-1238  CTTCTCCACCTGAGGCAAAGCCCCAAGGCTGGGTTGTTATCACTTGCCAATTGTCCAAAT
OM179883.1_52-1236  CTTCTCCACCTGAGGCAAAGCCCCAAGGCTGGGTTGTTATCACTTGCCAATTGTCCAAAT
OM256458.1_54-1238  CTTCTCCACCTGAGGCAAAGCCCCAAGGCTGGGTTGTTATCACTTGCCAATTGTCCAAAT
PQ530038.1_1-1185   CTTCTCCACCTGAGGCAAAGCCCCAAGGCTGGGTTGTTATCACTTGCCAATTGTCCAAAT
MZ274340.1_1-1185   CTTCTCCACCTGAGGCAAAGCCCCAAGGCTGGGTTGTTATCACTTGCCAATTGTCCAAAT
KF364615.2_14-1198  CTTCTCCACCTGAGGCAAAGCCCCAAGGCTGGGTTGTTATCACTTGCCAATTGTCCAAAT
MZ274341.1_1-1185   CTTCTCCACCTGAGGCAAAGCCCCAAGGCTGGGTTGTTATCACTTGCCAATTGTCCAAAT
KC915036.1_54-1238  CTTCTCCACCTGAGGCAAAGCCCCAAGGCTGGGTTGTTATCACTTGCCAATTGTCCAAAT
OR365552.1_54-1238  CTTCTCCACCTGAGGCAAAGCCCCAAGGCTGGGTTGTTATCACTTGCCAATTGTCCAAAT
OR365551.1_54-1238  CTTCTCCACCTGAGGCAAAGCCCCAAGGCTGGGTTGTTATCACTTGCCAATTGTCCAAAT
```

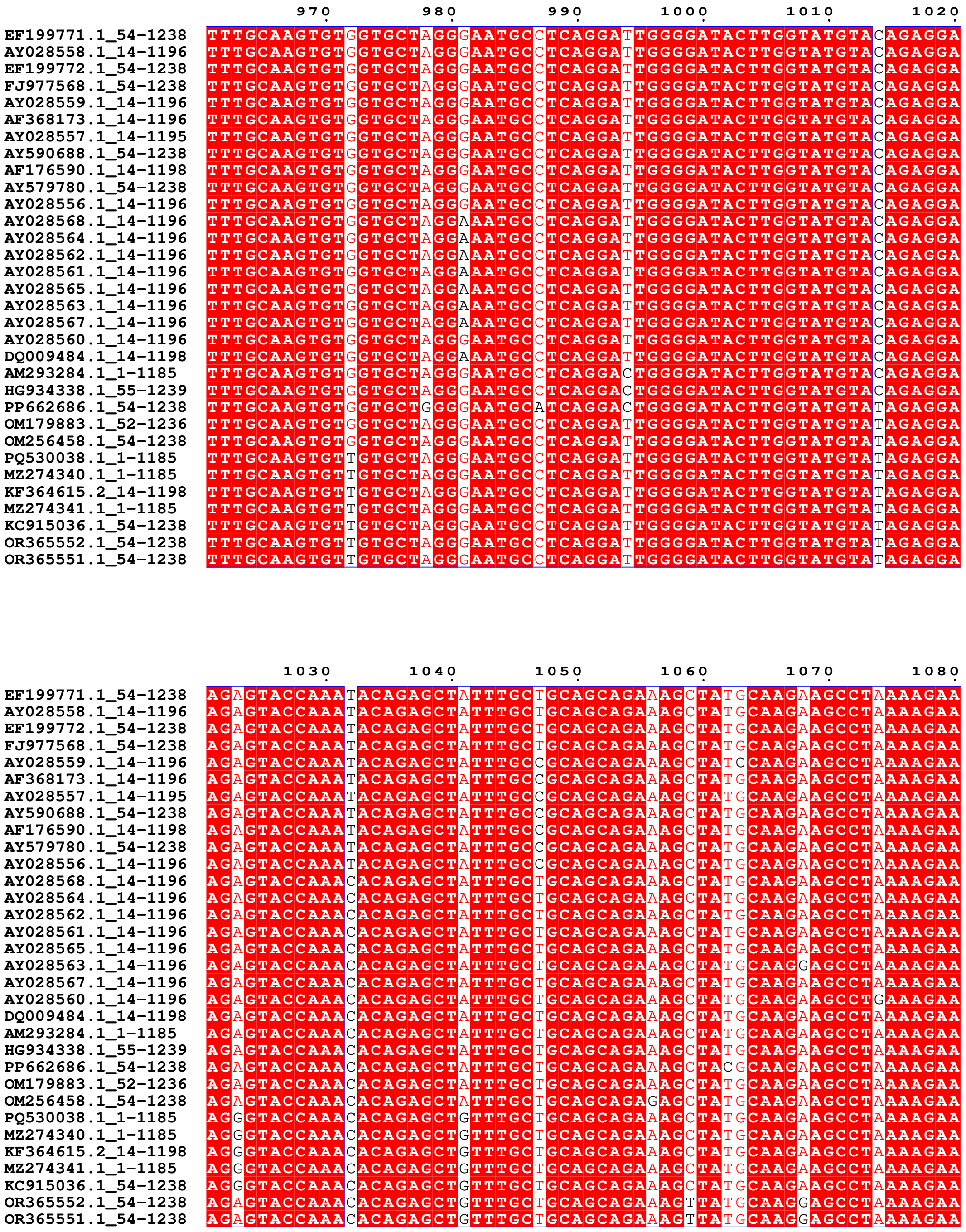
970 980 990 1000 1010 1020
EF199771.1_54-1238 TTTGCAAGTGTGGTGCTAGGGAATGCCTCAGGATTGGGGATACTTGGTATGTACAGAGGA
AY028558.1_14-1196 TTTGCAAGTGTGGTGCTAGGGAATGCCTCAGGATTGGGGATACTTGGTATGTACAGAGGA
EF199772.1_54-1238 TTTGCAAGTGTGGTGCTAGGGAATGCCTCAGGATTGGGGATACTTGGTATGTACAGAGGA
FJ977568.1_54-1238 TTTGCAAGTGTGGTGCTAGGGAATGCCTCAGGATTGGGGATACTTGGTATGTACAGAGGA
AY028559.1_14-1196 TTTGCAAGTGTGGTGCTAGGGAATGCCTCAGGATTGGGGATACTTGGTATGTACAGAGGA
AF368173.1_14-1196 TTTGCAAGTGTGGTGCTAGGGAATGCCTCAGGATTGGGGATACTTGGTATGTACAGAGGA
AY028557.1_14-1195 TTTGCAAGTGTGGTGCTAGGGAATGCCTCAGGATTGGGGATACTTGGTATGTACAGAGGA
AY590688.1_54-1238 TTTGCAAGTGTGGTGCTAGGGAATGCCTCAGGATTGGGGATACTTGGTATGTACAGAGGA
AF176590.1_14-1198 TTTGCAAGTGTGGTGCTAGGGAATGCCTCAGGATTGGGGATACTTGGTATGTACAGAGGA
AY579780.1_54-1238 TTTGCAAGTGTGGTGCTAGGGAATGCCTCAGGATTGGGGATACTTGGTATGTACAGAGGA
AY028556.1_14-1196 TTTGCAAGTGTGGTGCTAGGGAATGCCTCAGGATTGGGGATACTTGGTATGTACAGAGGA
AY028568.1_14-1196 TTTGCAAGTGTGGTGCTAGGAAATGCCTCAGGATTGGGGATACTTGGTATGTACAGAGGA
AY028564.1_14-1196 TTTGCAAGTGTGGTGCTAGGAAATGCCTCAGGATTGGGGATACTTGGTATGTACAGAGGA
AY028562.1_14-1196 TTTGCAAGTGTGGTGCTAGGAAATGCCTCAGGATTGGGGATACTTGGTATGTACAGAGGA
AY028561.1_14-1196 TTTGCAAGTGTGGTGCTAGGAAATGCCTCAGGATTGGGGATACTTGGTATGTACAGAGGA
AY028565.1_14-1196 TTTGCAAGTGTGGTGCTAGGAAATGCCTCAGGATTGGGGATACTTGGTATGTACAGAGGA
AY028563.1_14-1196 TTTGCAAGTGTGGTGCTAGGAAATGCCTCAGGATTGGGGATACTTGGTATGTACAGAGGA
AY028567.1_14-1196 TTTGCAAGTGTGGTGCTAGGAAATGCCTCAGGATTGGGGATACTTGGTATGTACAGAGGA
AY028560.1_14-1196 TTTGCAAGTGTGGTGCTAGGGAATGCCTCAGGATTGGGGATACTTGGTATGTACAGAGGA
DQ009484.1_14-1198 TTTGCAAGTGTGGTGCTAGGAAATGCCTCAGGATTGGGGATACTTGGTATGTACAGAGGA
AM293284.1_1-1185 TTTGCAAGTGTGGTGCTAGGGAATGCCTCAGGACTGGGGATACTTGGTATGTACAGAGGA
HG934338.1_55-1239 TTTGCAAGTGTGGTGCTAGGGAATGCCTCAGGACTGGGGATACTTGGTATGTACAGAGGA
PP662686.1_54-1238 TTTGCAAGTGTGGTGCTGGGGAATGCATCAGGACTGGGGATACTTGGTATGTATAGAGGA
OM179883.1_52-1236 TTTGCAAGTGTGGTGCTAGGGAATGCCTCAGGATTGGGGATACTTGGTATGTATAGAGGA
OM256458.1_54-1238 TTTGCAAGTGTGGTGCTAGGGAATGCCTCAGGATTGGGGATACTTGGTATGTATAGAGGA
PQ530038.1_1-1185 TTTGCAAGTGTTGTGCTAGGGAATGCCTCAGGATTGGGGATACTTGGTATGTATAGAGGA
MZ274340.1_1-1185 TTTGCAAGTGTTGTGCTAGGGAATGCCTCAGGATTGGGGATACTTGGTATGTATAGAGGA
KF364615.2_14-1198 TTTGCAAGTGTTGTGCTAGGGAATGCCTCAGGATTGGGGATACTTGGTATGTATAGAGGA
MZ274341.1_1-1185 TTTGCAAGTGTTGTGCTAGGGAATGCCTCAGGATTGGGGATACTTGGTATGTATAGAGGA
KC915036.1_54-1238 TTTGCAAGTGTTGTGCTAGGGAATGCCTCAGGATTGGGGATACTTGGTATGTATAGAGGA
OR365552.1_54-1238 TTTGCAAGTGTTGTGCTAGGGAATGCCTCAGGATTGGGGATACTTGGTATGTATAGAGGA
OR365551.1_54-1238 TTTGCAAGTGTTGTGCTAGGGAATGCCTCAGGATTGGGGATACTTGGTATGTATAGAGGA
1030 1040 1050 1060 1070 1080
EF199771.1_54-1238 AGAGTACCAAATACAGAGCTATTTGCTGCAGCAGAAAGCTATGCAAGAAGCCTAAAAGAA
AY028558.1_14-1196 AGAGTACCAAATACAGAGCTATTTGCTGCAGCAGAAAGCTATGCAAGAAGCCTAAAAGAA
EF199772.1_54-1238 AGAGTACCAAATACAGAGCTATTTGCTGCAGCAGAAAGCTATGCAAGAAGCCTAAAAGAA
FJ977568.1_54-1238 AGAGTACCAAATACAGAGCTATTTGCTGCAGCAGAAAGCTATGCAAGAAGCCTAAAAGAA
AY028559.1_14-1196 AGAGTACCAAATACAGAGCTATTTGCCGCAGCAGAAAGCTATCCAAGAAGCCTAAAAGAA
AF368173.1_14-1196 AGAGTACCAAATACAGAGCTATTTGCCGCAGCAGAAAGCTATGCAAGAAGCCTAAAAGAA
AY028557.1_14-1195 AGAGTACCAAATACAGAGCTATTTGCCGCAGCAGAAAGCTATGCAAGAAGCCTAAAAGAA
AY590688.1_54-1238 AGAGTACCAAATACAGAGCTATTTGCCGCAGCAGAAAGCTATGCAAGAAGCCTAAAAGAA
AF176590.1_14-1198 AGAGTACCAAATACAGAGCTATTTGCCGCAGCAGAAAGCTATGCAAGAAGCCTAAAAGAA
AY579780.1_54-1238 AGAGTACCAAATACAGAGCTATTTGCCGCAGCAGAAAGCTATGCAAGAAGCCTAAAAGAA
AY028556.1_14-1196 AGAGTACCAAATACAGAGCTATTTGCCGCAGCAGAAAGCTATGCAAGAAGCCTAAAAGAA
AY028568.1_14-1196 AGAGTACCAAACACAGAGCTATTTGCTGCAGCAGAAAGCTATGCAAGAAGCCTAAAAGAA
AY028564.1_14-1196 AGAGTACCAAACACAGAGCTATTTGCTGCAGCAGAAAGCTATGCAAGAAGCCTAAAAGAA
AY028562.1_14-1196 AGAGTACCAAACACAGAGCTATTTGCTGCAGCAGAAAGCTATGCAAGAAGCCTAAAAGAA
AY028561.1_14-1196 AGAGTACCAAACACAGAGCTATTTGCTGCAGCAGAAAGCTATGCAAGAAGCCTAAAAGAA
AY028565.1_14-1196 AGAGTACCAAACACAGAGCTATTTGCTGCAGCAGAAAGCTATGCAAGAAGCCTAAAAGAA
AY028563.1_14-1196 AGAGTACCAAACACAGAGCTATTTGCTGCAGCAGAAAGCTATGCAAGGAGCCTAAAAGAA
AY028567.1_14-1196 AGAGTACCAAACACAGAGCTATTTGCTGCAGCAGAAAGCTATGCAAGAAGCCTAAAAGAA
AY028560.1_14-1196 AGAGTACCAAACACAGAGCTATTTGCTGCAGCAGAAAGCTATGCAAGAAGCCTGAAAGAA
DQ009484.1_14-1198 AGAGTACCAAACACAGAGCTATTTGCTGCAGCAGAAAGCTATGCAAGAAGCCTAAAAGAA
AM293284.1_1-1185 AGAGTACCAAACACAGAGCTATTTGCTGCAGCAGAAAGCTATGCAAGAAGCCTAAAAGAA
HG934338.1_55-1239 AGAGTACCAAACACAGAGCTATTTGCTGCAGCAGAAAGCTATGCAAGAAGCCTAAAAGAA
PP662686.1_54-1238 AGAGTACCAAACACAGAGCTATTTGCTGCAGCAGAAAGCTACGCAAGAAGCCTAAAAGAA
OM179883.1_52-1236 AGAGTACCAAACACAGAGCTATTTGCTGCAGCAGAAAGCTATGCAAGAAGCCTAAAAGAA
OM256458.1_54-1238 AGAGTACCAAACACAGAGCTATTTGCTGCAGCAGAGAGCTATGCAAGAAGCCTAAAAGAA
PQ530038.1_1-1185 AGGGTACCAAACACAGAGCTGTTTGCTGCAGCAGAAAGCTATGCAAGAAGCCTAAAAGAA
MZ274340.1_1-1185 AGGGTACCAAACACAGAGCTGTTTGCTGCAGCAGAAAGCTATGCAAGAAGCCTAAAAGAA
KF364615.2_14-1198 AGGGTACCAAACACAGAGCTGTTTGCTGCAGCAGAAAGCTATGCAAGAAGCCTAAAAGAA
MZ274341.1_1-1185 AGGGTACCAAACACAGAGCTGTTTGCTGCAGCAGAAAGCTATGCAAGAAGCCTAAAAGAA
KC915036.1_54-1238 AGGGTACCAAACACAGAGCTGTTTGCTGCAGCAGAAAGCTATGCAAGAAGCCTAAAAGAA
OR365552.1_54-1238 AGAGTACCAAACACAGAGCTGTTTGCTGCAGCAGAAAGTTATGCAAGGAGCCTAAAAGAA
OR365551.1_54-1238 AGAGTACCAAACACAGAGCTGTTTGCTGCAGCAGAAAGTTATGCAAGGAGCCTAAAAGAA

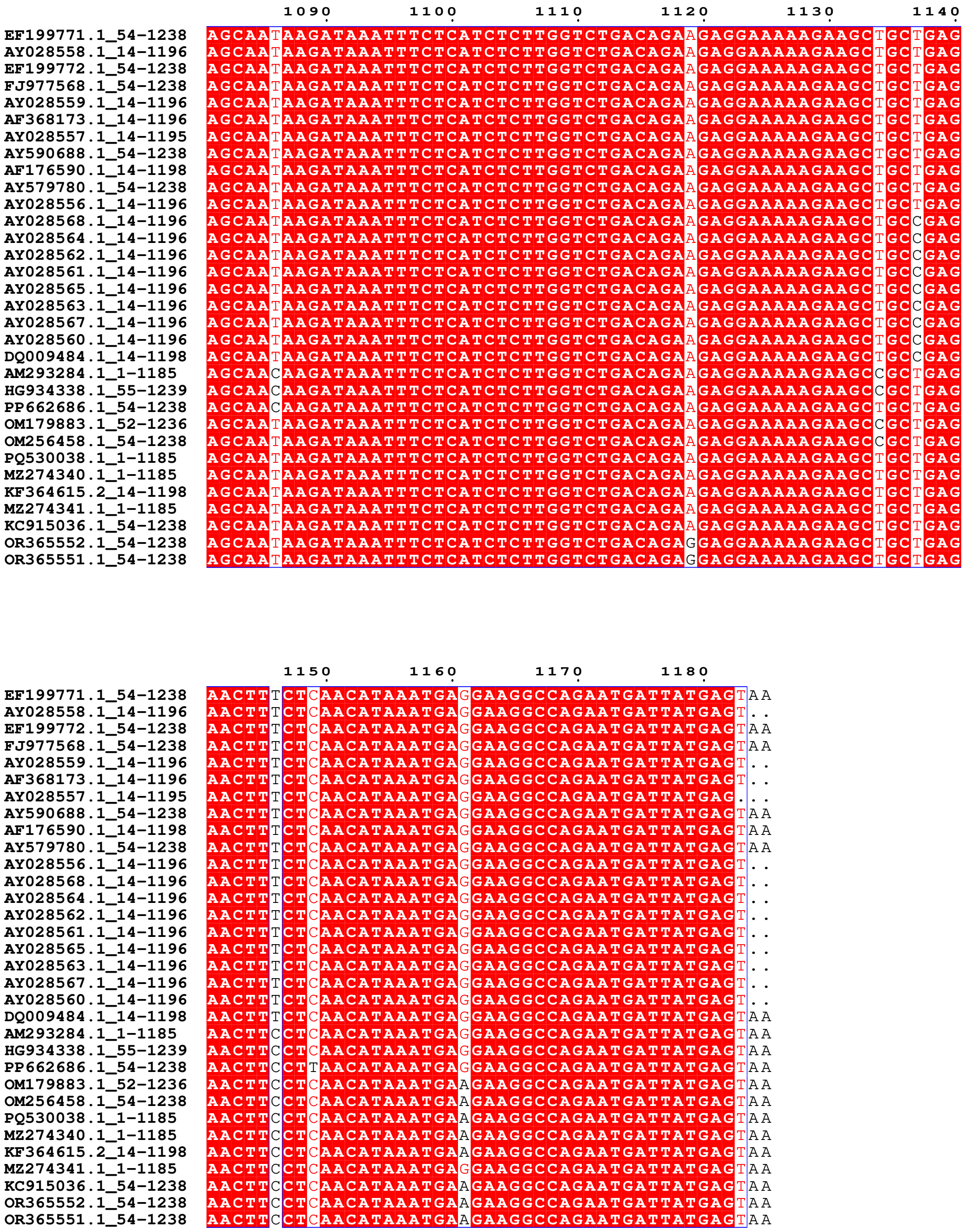

Figure S1. in silico conservation analysis
